# Leveraging the Metabolic Fingerprint of Sleep Deprivation and Sleep Restriction for Forensic Applications: A Machine Learning Study in Oral Fluid Metabolomics

**DOI:** 10.1021/acs.jproteome.5c01064

**Published:** 2026-05-06

**Authors:** Michael Scholz, Andrea E. Steuer, Akos Dobay, Hans-Peter Landolt, Thomas Kraemer

**Affiliations:** † Department of Forensic Pharmacology and Toxicology, Zurich Institute of Forensic Medicine, University of Zurich, Zurich 8006, Switzerland; ‡ Forensic Machine Learning Technology Center, University of Zurich, Zurich 8006, Switzerland; § Institute of Pharmacology and Toxicology, University of Zurich, Zurich 8057, Switzerland; ∥ Sleep & Health Zurich, University of Zurich, Zurich 8008, Switzerland

**Keywords:** metabolic fingerprint, sleep deprivation, sleep restriction, forensic, machine learning, oral fluid, metabolomics, LC-HRMS, classification, sleep loss

## Abstract

As sleep loss leads to accidents and impaired safety, a direct metabolic marker would be beneficial for forensic interpretation. In a sufficiently powered, randomized, controlled, crossover trial under realistic conditions, we examined the salivary metabolome of 20 young men (habitual sleep duration 7–9 h) following three interventions: one night of total sleep deprivation, four consecutive nights of sleep restriction to 6 h, and control (8 h of sleep). Oral fluid specimens were repeatedly collected and analyzed using liquid chromatography coupled to mass spectrometry. Logistic regression models were trained to classify unseen samples without reference samples from the same individual. Acute sleep deprivation exhibited a unique metabolic fingerprint that could be detected precisely (F_0.5_ = 0.90) when using only 12 molecular features. This fingerprint was more pronounced in samples collected in the morning/midday hours. Nevertheless, at all time points, the overall correct predictions by far outweighed the incorrect ones. Four nights of sleep restriction did not lead to exploitable metabolic changes. This study presents a metabolic fingerprint of acute sleep deprivation in oral fluid under realistic conditions and explores practical implications and limitations of its machine learning-aided classification. Metabolomics-based, reference-free sleep loss detection holds potential for applications in forensic, clinical, and occupational contexts.

## Graphical abstract

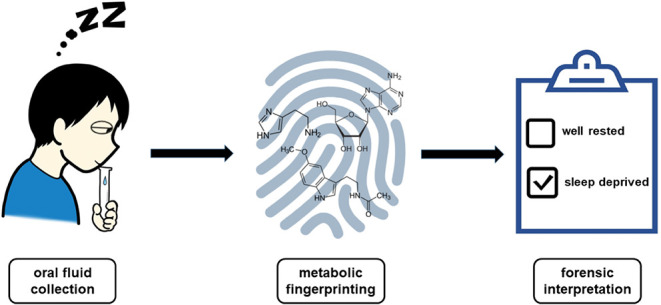

## Introduction

Sleep loss, a multifaceted and ever-growing problem in modern societies, leads to high costs by affecting public health, productivity, general well-being, and traffic safety.^1,2^ It can manifest itself in the form of either shortage of sleep (i.e., sleep restriction) or total sleep deprivation. Despite its widespread prevalence, there is no simple, direct, objective, and reliable measurement.^3^ In fact, to date, forensic professionals must rely on self-reports to detect sleep loss. A robust test would not be limited to road safety but would have numerous applications across sectors where alertness and cognitive function are critical to performance and safety.^4^

In the past, basic research has tried to identify metabolic changes that occurred under both acute and chronic sleep loss.^5–8^ Proteins that play an important role in the immune system (e.g., interleukin-6 and interleukin-1 beta, tumor necrosis factor alpha, C-reactive protein, or salivary amylase) have been investigated as possible markers based on a dysregulated immune response after sleep loss.^9–16^ Other works could detect metabolic changes after sleep deprivation.^17–25^ However, none of these biomarkers met the requirements for the detection of acute sleepiness or for posthoc forensic analysis due to their lack of specificity for sleep loss, real-world constraints, or the need for an additional reference sample, which is usually not available in practical applications. Moreover, most of these studies applied constant routine laboratory protocols (e.g., controlled posture, activity, nutrient intake, and lighting), thus excluding environmental and behavioral variables present in real-world conditions from the outset.

Another challenge, particularly for roadside or point-of-care (POC) testing, is the selection of the appropriate biological matrix, as blood draws can be inconvenient or legally prohibited for non-medical personnel due to their invasive nature. Owing to its quick, simple, cheap, and non-invasive sampling procedure, as well as its widespread use for drug testing in many countries around the globe, we identified oral fluid (OF) as the ideal biofluid to meet these requirements.^26–28^

Metabolomics is the study of small molecules present in biological samples, representing the functional end points of cellular processes. In forensic science, it allows for the measurement of metabolic changes under various toxicological conditions.^29^ For instance, forensic applications include estimating postmortem interval,^30,31^ determining cause of death,^32,33^ and identifying novel biomarkers.^34,35^ Metabolomics analyses are increasingly combined with machine learning techniques to process the complex and high-dimensional data for classification, regression, and pattern recognition tasks. This linkage enables identification of metabolic fingerprints and improves the accuracy of forensic determinations by processing vast amounts of metabolic data that would be impossible to analyze manually.

We hypothesized that a metabolic fingerprint detectable in OF could demonstrate sleep loss above a certain threshold. After recovery sleep, this shift should disappear, illustrating its sleep dependency.^36^ In this context, we conducted a clinical trial to examine the metabolic changes that arise from varying levels of sleep loss under realistic conditions, using study interventions that replicated the most common sleep-wake scenarios.^37^ For this first-of-its-kind study, we focused on young men, who constitute the highest-risk group for sleepiness-induced road traffic accidents.^38–41^

The aim of this exploratory investigation was therefore to characterize the OF metabolome after different levels of sleep loss in a controlled but realistic setting. By leveraging interpretable machine learning techniques, classification models for sleep loss detection should be generated and evaluated from a forensic perspective. We thus explore the theoretical potential and limitations of reference-free metabolomics-based sleep loss detection.

## Experimental Section

### Study Protocol and Cohort

The study was approved by the local ethics committee (Kantonale Ethikkommission Zürich, reference number 2022-01273) and conducted in accordance with the Declaration of Helsinki of 1964 and its later amendments. Written informed consent was obtained from all participants. The full protocol has been preregistered (ClinicalTrials.gov ID NCT05585515) and published elsewhere.^37^ In this manuscript, we report the primary outcome of this trial, namely, changes in metabolite concentrations in oral fluid quantified by liquid chromatography with mass spectrometry. In brief, we applied three different interventions in a randomized, crossover fashion – namely sleep deprivation (SD), sleep restriction (SR), and control (C) – which were based on highly prevalent real-life sleep/wake scenarios. In the control condition, participants spent 8 h of time-in-bed at home prior to the first experimental night in the sleep laboratory. In both the SR and the SD conditions, an equal sleep deficit of 8 h was accumulated by either shortening habitual time-in-bed by 2 h over four consecutive nights (SR) or skipping a total night of sleep (SD). The three experimental conditions occurred in random order and were at least 1 week apart. The median study duration for a participant (beginning of first condition until end of third condition) was 26 days; the longest was 46 days. The study team closely monitored adherence to the protocol and the behavioral instructions, ensuring no opportunities for microsleeps or nap episodes. Time-in-bed was verified by actigraphy (GENEActiv, Activinsights Ltd., Kimbolton, United Kingdom) and completion of sleep-wake diaries. Importantly, confounding factors known to interfere with sleep-wake physiology and the metabolome were either eliminated (e.g., poor health status) or strictly controlled (e.g., extreme temperatures, nocturnal light, and nutrition) while ensuring an environment close to real life, with no restrictions on participants’ habitual movements or access to daylight.^37^

As documented in the power calculation based on our pilot study, a sample size of 17 participants was minimally required to detect significant effects (α = 5%, β = 20) of the interventions on the primary outcome variable (changes in the metabolic profile in oral fluid).^37^

We enrolled 29 volunteers, of whom 9 had to be excluded due to time constraints (n = 5), study criteria (n = 3), or loss of contact (n = 1). Of the remaining, 17 completed all three study interventions, and 3 partially completed (see consort diagram in Figure S1). All participants were healthy, young men of normal weight who reported a habitual sleep duration between 7 and 9 h per night, good sleep quality and no extreme chronotypes. The study protocol contained additional inclusion and exclusion criteria for secondary outcomes. The demographic characteristics of the study participants are presented in Table 1. This trial’s data collection took place from November 2022 to May 2023 (from the first person’s first visit to the last person’s last visit).

**Table 1:** Demographic characteristics of study participants (n = 20)^a^

	Mean ± standard deviation	Median (range)
Age [years]	24.0 ± 3.5	22.4 (20.0–33.3)
Body Mass Index [kg/m^2^]	21.3 ± 1.7	21.2 (19.1–24.9)
Diurnal preference	14.4 ± 1.7	14 (12–17)
Sleep quality	2.9 ± 1.5	3 (0–5)
Sleep efficacy [%]	92.1 ± 2.9	92.0 (86.9–98.2)

a The characteristics were measured as follows. Age: at first day of first intervention. Diurnal preference: reduced Morningness-Eveningness-Questionnaire (rMEQ) score.^42,43^ Sleep quality: Pittsburgh Sleep Quality Index (PSQI).^44^ We used validated German translations of the rMEQ and the PSQI. Sleep efficacy: polysomnographically recorded total sleep time divided by time-in-bed (8 h) in the screening night.

### Oral Fluid Collection

In each condition, eight OF samples of participants were collected at predefined clock times, as shown in Figure S2 and Text S1. One specimen was retrieved before the last night of each intervention schedule (t_1_: 19:00, baseline), six specimens after each intervention schedule (t_2_: 08:00, t_3_: 10:00, t_4_: 12:00, t_5_: 16:00, t_6_: 19:00, t_7_: 23:00), and one specimen after the recovery nights (t_8_: 08:00). For melatonin quantification, four additional OF samples were collected hourly between 20:00 and 23:00 (m_1_–m_4_), and two further samples were harvested at 00:00 and 01:00 (m_5_, m_6_) during the SR and SD conditions. Participants were not allowed to eat, drink, or toothbrush 30 min before collection procedures, but water-rinsed their mouths 10 min before. They placed a Salivette swab (Sarstedt AG, Sevelen, Switzerland) below the tongue for 2 min without chewing to ensure the collection of unstimulated OF.^45,46^ Upon sample collection, swabs were immediately centrifuged (3 min at 3,000 *g*) and stored in aliquots of 300 μL at −80 °C until analyzed.

### Dim-Light Melatonin Onset Estimation

Melatonin quantification in destined OF samples was performed via targeted liquid chromatography (LC) coupled to tandem mass spectrometry (MS/MS) analysis within a week after collection. Sample concentrations were calculated with a five-point external calibration curve in the range 1, 10, 25, 50, and 100 pg/mL for melatonin in water with the given internal standard concentration (peak area ratio to internal standard, unweighted curve). The detailed sample preparation protocol and instrumental settings are provided in Text S2 and Tables S1–S3. For dim-light melatonin onset (DLMO) estimation, we used the hockey-stick method, which fits two functions, a straight line and a parabola, to the evening melatonin profile.^47^ Their intersection is called the inflection point and serves as a reliable estimate of the DLMO time.^48^ Each participant’s DLMO time was assessed after each study run and used for normalization of metabolite profiles to individual circadian time. With this information, cosinor analysis was performed to evaluate rhythmicity of metabolites depending on DLMO time, using CosinorPy Python package (version 3.1).^49^

### Metabolic Fingerprinting

An untargeted metabolomics analysis was performed via LC coupled to high-resolution MS (i.e., LC-HRMS), using a Thermo Fisher Ultimate 3000 UHPLC system (Thermo Fisher Scientific, San Jose, CA, USA) coupled to a TripleTOF 6600 (Sciex, Concord, Ontario, Canada), as described in our published workflow.^50^ Details of the sample preparation and the instrumental settings can be found in Text S3 and Tables S4–S5. All samples were measured in four different LC-MS modes (reversed-phase chromatography with positive and negative ionization, RP+ and RP–, and hydrophilic interaction liquid chromatography with positive and negative ionization, HILIC+ and HILIC−) to cover the broad chemical diversity of metabolites in OF. MS-DIAL software (version 4.9.221218) was used for raw MS data processing (peak picking, deconvolution, alignment).^51^ Detailed parameter settings are presented in Table S6. Critical parameters were optimized beforehand with Paramounter R program in a subset analysis of pooled quality control sample data.^52^ We revised and, if necessary, corrected all peak integrations manually and thoroughly if a given signal turned out to be of importance in the subsequent data analysis process. After every manual correction step, the entire data analysis pipeline was rerun. This cycle was repeated until every important signal was revised or corrected. The resulting tables of molecular features (MFs) and corresponding peak areas per LC-MS condition were exported, and subsequent data preprocessing was performed in Python (version 3.12.3). The peak area data underwent batch and run-order correction, filtering by measurement robustness criteria, normalization, transformation, scaling, and missing data imputation, according to the parameters and packages mentioned in Text S4.

Linear discriminant analysis (LDA) was performed to assess the ability of molecular features to distinguish between the different study conditions. Analyses were conducted separately on the HILIC and RP data sets, as well as on the combined data set, to elucidate the metabolic characteristics driving group separation based on explained variance ratios and Fisher criterion. All computations were performed using the LinearDiscriminantAnalysis module from scikit-learn (version 1.5.0).^53^

### Classification Model Training

Logistic regression algorithm (from scikit-learn module LogisticRegression) was used for building classification models to determine whether an unseen test sample belonged to the sleep-deprived, sleep-restricted, or rested condition of a study participant. Before any selection or model-building step, the untargeted metabolomics data set was split into a training data set (for model fitting and hyperparameter optimization) and a test data set (for scoring of relevant metrics to evaluate the performance and generalization ability of the trained model). The split was done subject-wise on complete data sets, generating a training set size of 18 subjects’ data sets (fully and partly completed, 89% of all data points) and a test set size of 2 subjects’ data sets (fully completed, 11% of all data points) using scikit-learn LeavePGroupsOut module (parameter: n_groups = 2). All incomplete data sets were kept in the training set. This split procedure and all subsequent data analysis steps were run on all possible train/test combinations separately (thus building 136 independent models after 136 independent splits) to generate a robust and generalizable performance estimate. During all procedures, k-fold cross-validation (CV) was applied, using a stratified group k-fold splitter (scikit-learn module StratifiedGroupKFold, assigning subjects’ aliases as groups, i.e., stratification by participant) that ensures that the samples of the same subject are not split into the training subset and the validation subset which prevents overly optimistic estimation performances. In all models with comparisons with the sleep deprivation condition, the SD condition was labeled as positive case (binary code 1). Further, the control condition was always labeled as negative case (binary code 0).

Model training consisted of a nested cross-validation loop approach, including hyperparameter optimization and recursive feature elimination, as specified in Figures S3–S4, Text S5, and Table S7.^54^ To remove non-robust or non-sleep-related features before entering the nested CV loop, we applied an analysis of variance (ANOVA) filter on each training data set separately, excluding MF that showed significant differences between the study conditions at time points t_1_ or t_8_ (before the sleep intervention or after the recovery night; MF removed if F-value < 0.05).^36^ The models were trained on the processed molecular feature table of the time points t_2_–t_7_ of the training data set (between intervention night and recovery night), when effects of the interventions were expected.

### Model Evaluations and Optimization for Forensic Applications

After application of the optimization and feature elimination procedures, the feature table was reduced to an acceptable number of features (for roadside or POC testing), and each model’s decision abilities were post-tuned by gradually changing the threshold of the classifier’s decision function, using scikit-learn FixedThresholdClassifier module and evaluation of the final classification metrics.^55^ Given that specific applications of decisive models require distinct decision thresholds, we post-tuned the threshold for the probability estimates of the classifiers for the forensic use case, where false positive results are deemed more harmful than false negatives since they could lead to prosecution of innocent people. Therefore, we evaluated our model with the F_0.5_ score, which is derived from the F_1_ score but penalizes false positives errors twice as much as false negative errors (see Equation S1). Only for comparison with other studies but not of interest in the forensic use case, we also computed the corresponding F_1_ scores, accuracies, and Matthews correlation coefficient (MCC) values.

### Sleep Dependency and Model Sanity

The models were tested for their sleep dependency, and a permutation study was conducted. In the sleep dependency test, the final reduced and tuned models were meant to predict the study samples that were collected before the actual study interventions (at time point t_1_ for C and SD interventions), and after the recovery nights of the participants (at time point t_8_ for all interventions). Finally, yet importantly, a permutation test of the optimized classifiers was conducted (scikit-learn module permutation_test_score, parameters: cv = 8, n_permutations = 1000). This permutation test was performed 1000 times using 8-fold cross-validation (i.e., 8000 iterations) on each model and the mean classification metrics, their standard deviations, and the p-values collected.

## Results and Discussion

### DLMO Estimation

We could compute the dim-light melatonin onset (DLMO) times in 54 out of 55 evening melatonin profiles. The median DLMO time was 21:41 (IQR 20:56–22:14). An overview of each participant’s DLMO estimates is presented in Figure S5.

### Metabolic Fingerprinting

In total, 440 OF samples from 20 different donors were analyzed for their metabolic content in the untargeted metabolomics analysis. After data preprocessing, the combined feature table covered 6035 robust molecular features (2173, 668, 2993, 201, from HILIC+, HILIC–, RP+, and RP– modes, respectively).

Separate LDA result plots of the HILIC, RP, and the full data set after sleep interventions where applied are portrayed in Figure 1. Based on visual separation, the explained variance ratios along the LD component axes, and the Fisher criterion results, the different study interventions form clusters to a certain degree. Overall, there appears to be a poor discrimination between the control and the sleep restriction condition. More specifically, in the full data set, the large overlap along the second LD component axis accounts for only 18.3% of the variance between the classes. By contrast, the metabolic characteristics of oral fluid after a night of sleep deprivation clearly differed from the control and SR conditions along the first LD component axis, explaining 81.7% of the total variance. The separation increases (higher Fisher criterion) with lower number of features (i.e., less dimensions) and is mostly driven by the more hydrophilic metabolites from the HILIC data set.

**Figure 1:**
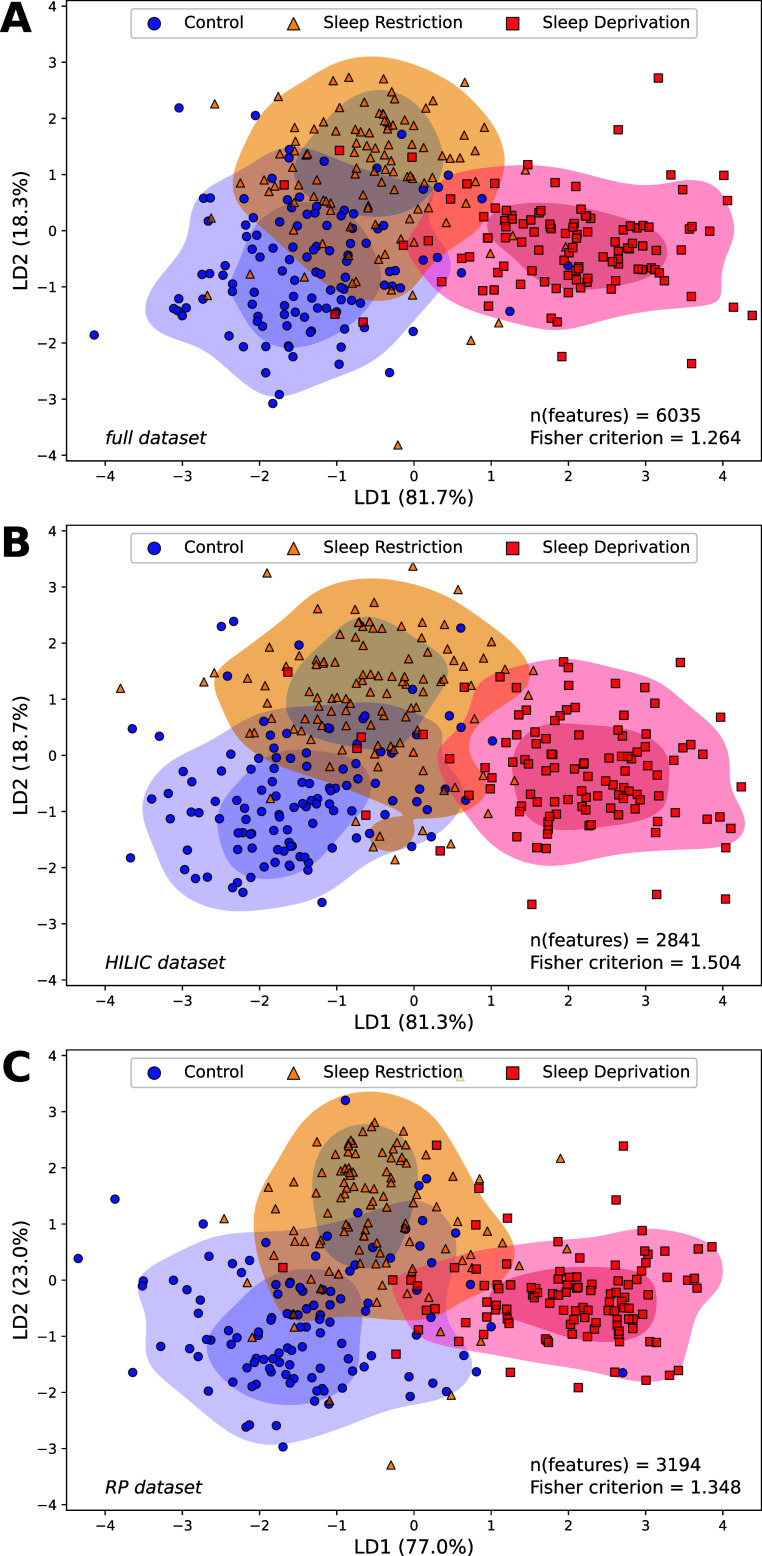
LDA scatter plots of all 330 study samples taken between time points t_2_–t_7_, grouped by study interventions. A. Full data set consisting of 6035 molecular features. B. HILIC data set consisting of 2841 molecular features. C. Reversed-phase (RP) data set consisting of 3194 molecular features. Each body on the plot represents an individual sample, positioned according to its values on the first two discriminant components. Samples from control intervention as blue circles, from sleep restriction intervention as orange triangles, and from sleep deprivation intervention as red squares. Kernel density estimates around each intervention mean in two shades of the respective color coding.

### Classification Models

Consequently, we focused on building three final classification models that can discriminate the sleep deprivation condition

(I) from the control condition (SD vs C model), and
(II) from the sleep restriction condition (SD vs SR model), and
(III) from the control and sleep restriction conditions at once (SD vs C and SR model, i.e., overall SD classification).

While the one-vs-one classification models (I and II) primarily serve as theoretical limit estimates of the strength of sleep deprivation effects, the one-vs-rest model (III) has important practical relevance, since forensic experts must differentiate between various possible impairment states.

In fact, the metabolomics-based discrimination between sleep-restriction and well-rested (control) conditions remains elusive based on our data, especially when considering the high requirements for robustness and reliability in forensic analyses.

### Model Evaluations and Optimization for Forensic Applications

The results of the looped randomized hyperparameter search with recursive feature elimination for logistic regression model optimization and feature shrinkage are portrayed in Figure 2 and are extended in Figures S6–S8 and Text S6. The characteristics and performance metrics for the three classification models are summarized in Table 2. Comparing SD with C (model I) and SR (model II), the optimal models resulted in an area under the receiver operator characteristic curve (AUROC) of 0.92 and 0.85 for 656 and 131 features used, respectively. The optimal overall SD model (III) resulted in an AUROC of 0.90 using 1701 features.

**Table 2:** Overview of Characteristics and Performance Metrics of the Trained Classification Models^a^

	SD vs C	SD vs SR	SD vs C & SR
	(I)	(II)	(III)
Input features	6035	6035	6035
**AUROC** maximum (n features)	0.92 (656)	0.85 (131)	0.90 (1701)
**AUROC** in reduced set (n features)	0.86 (10)	0.80 (10)	0.86 (12)
**F**_**0.5**_ (decision threshold)	0.89 (0.70)	0.87 (0.65)	0.90 (0.65)
**Precision** (decision threshold)	0.96 (0.70)	0.96 (0.65)	0.94 (0.65)
**Recall** (decision threshold)	0.70 (0.70)	0.65 (0.65)	0.77 (0.65)
**F**_**1**_ (decision threshold)	0.81 (0.70)	0.77 (0.65)	0.85 (0.65)
**Accuracy** (decision threshold)	0.83 (0.70)	0.81 (0.65)	0.91 (0.65)
**MCC** (decision threshold)	0.69 (0.70)	0.65 (0.65)	0.79 (0.65)

a The decision threshold was tuned in each model in order to yield a maximum F_0.5_ score while using the reduced set of features.

**Figure 2:**
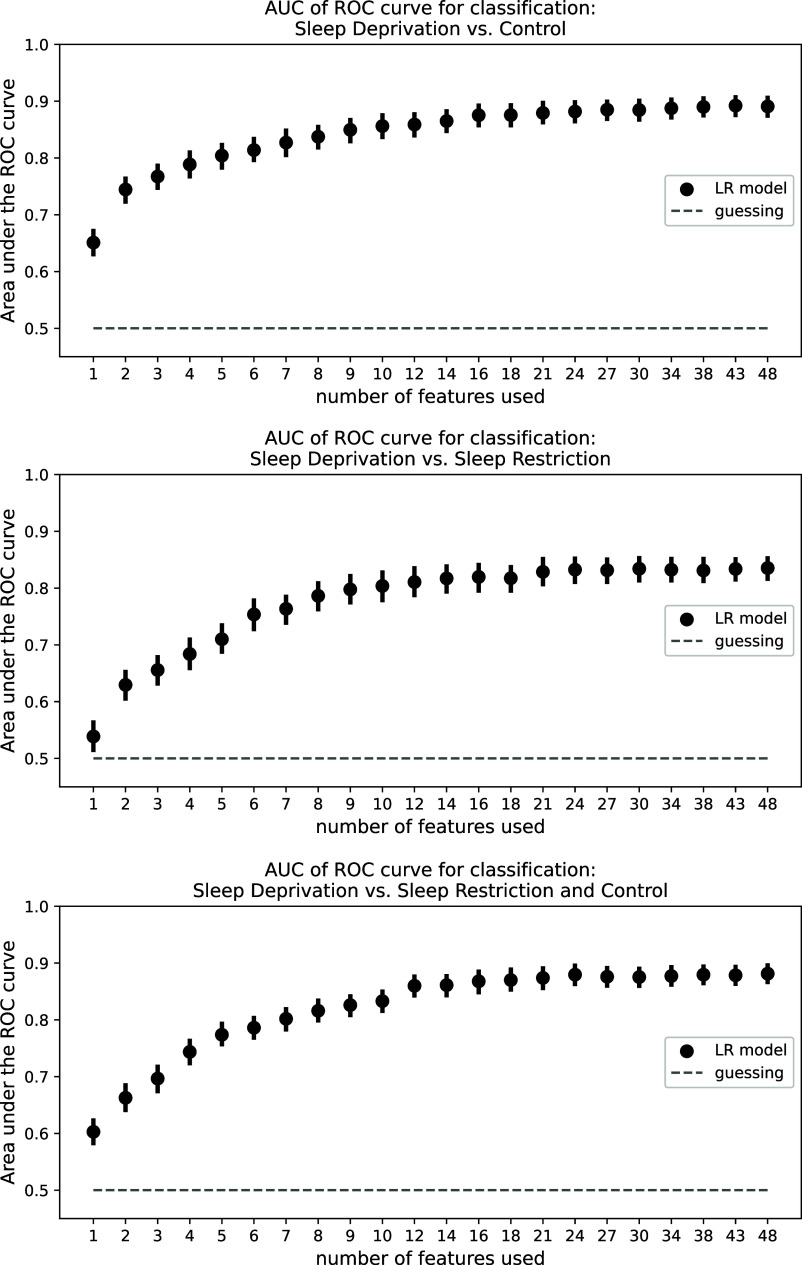
Graphical results of the looped randomized hyperparameter search with recursive feature elimination. Results for sleep deprivation vs control intervention classification (top), for sleep deprivation vs sleep restriction intervention classification (center), and for sleep deprivation vs control and sleep restriction intervention classification (bottom). Visualization of area under the receiver operator characteristic curve (AUROC) against number of features used. Dots with line represent logistic regression (LR) model mean with 95% confidence interval. Dashed line at AUROC = 0.5 represents random guessing model (current benchmark).

From an operator’s perspective, a decision must be made that considers the model’s applicability (which usually means having a small feature number) while maintaining acceptable classification power (high AUROC). For a laboratory test, an applicable number of features to test can be a few dozen, whereas a roadside test ideally contains as few analytes as possible. To enable roadside or POC testing, we aimed for the lowest feasible number of features that ensure reliable prediction performance. Based on the bend in the AUROC curves (Figure 2; i.e., begin of a ceiling effect), the feature table was shrunk to the best 10 features for SD vs C classification (AUROC = 0.86) and for SD vs SR classification (AUROC = 0.80), respectively. For the more complex overall SD classification, the feature table was reduced to the best 12 molecular features (AUROC = 0.86).

In other words, acute sleep deprivation of 8 h led to a unique metabolic fingerprint that was already detectable in a small fraction of the data set. These changes were most evident when compared with the rested control (C) intervention, but also, albeit to a lesser degree, when compared with the repeated sleep restriction (SR) intervention that shared an equal amount of total sleep loss. As expected from the LDA results, with an equal amount of training data, the SD vs C classification (model I) performance was slightly better than the SD vs SR classification (model II) performance. This difference seems logical given that in the sleep restriction condition, participants accumulated a sleep debt of 8 h and are thus in a state that is more similar to SD than the control condition with no sleep debt. Logically, the more complex one-vs-rest classification model (SD vs C and SR, model III) benefitted from two more features than the separate one-vs-one classifiers to achieve comparable performance.

The results of the decision threshold post-tuning and its implications for the classification metrics are visualized in Figure 3 and summarized in Table 2. In essence, when our models reported a positive outcome (sleep-deprived donor) for an unseen sample, it was correct 96% (against C or SD) or 94% (against both) of the time. Simultaneously, our models could correctly identify 70% (vs C), 65% (vs SR), and 77% (vs C and SR) of all study samples where the donor was actually sleep-deprived.

**Figure 3:**
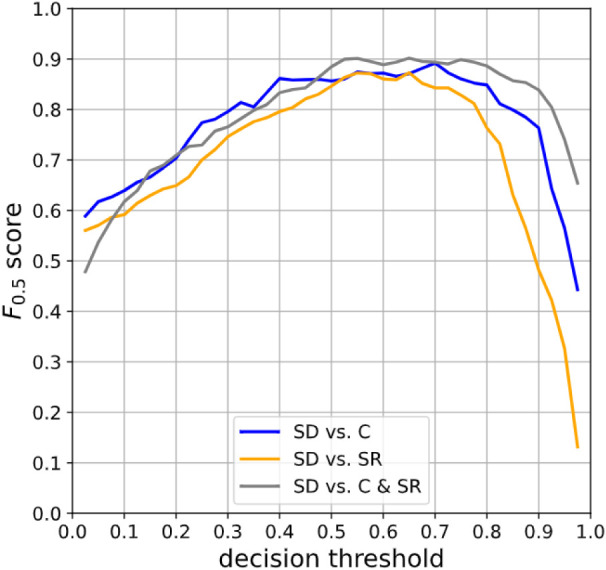
Results of post-threshold tuning for different models. The F_0.5_ scores achieved with different levels of the decision threshold are visualized for the SD vs C model (based on 10 features, blue line), the SD vs SR model (based on 10 features, orange line), and the overall SD model (based on 12 features, gray line).

Considering that such a test might imply legal consequences in the case of a positive result, the requirements are high, especially concerning false positive rates. Therefore, in the forensic use case of this predictive analysis for the detection of sleep deprivation, we suggest elevating the decision threshold to at least 0.70 for best result reliability. In other applications (e.g., in a clinical setting), a different threshold may be preferred for greater accuracy of results at the cost of more false positives. The final scores prove a satisfying performance of our models, especially when considering that the status quo is not better than random guessing (i.e., having no biomarkers).

The overall correct predictions of the models far outweighed the incorrect ones at all time points. Among the few misclassifications, it is worth noting that all models made more prediction errors toward the evening samples. This was reflected in the differences between the prediction probabilities, i.e., the prediction margins were smaller toward the later samples, especially at 23:00, as shown in Figure 4. These time-dependent performance fluctuations are in accordance with our pilot study, in which we demonstrated that the metabolic changes after sleep deprivation were most pronounced in the morning hours and concluded that the detection of SD in oral fluid would be most promising then.^36^ The present study confirmed this notion. The models showed large prediction margins in the samples collected between 08:00 and 12:00, while closer margins were present toward the evening, especially at 23:00. The differences may be explained by co-occurring sleep homeostatic and circadian processes that, under entrained conditions, temporarily counteract each other during the wake maintenance zone and may explain the relatively poor performance of the SD vs SR classifier in the later evening.^56,57^ In addition, the metabolomic findings are consistent with the notion that homeostatic sleep drive is not increasing linearly but according to a saturating exponential function.^58,59^ The predictive power of our models suggests that this principle is also reflected in the oral metabolome. Therefore, it cannot be expected that there is a single biomarker for sleepiness or SD, which increases linearly with hours of wakefulness. It is much more likely that distinct metabolites may interact to define the metabolic fingerprint of sleepiness at a given time.

**Figure 4:**
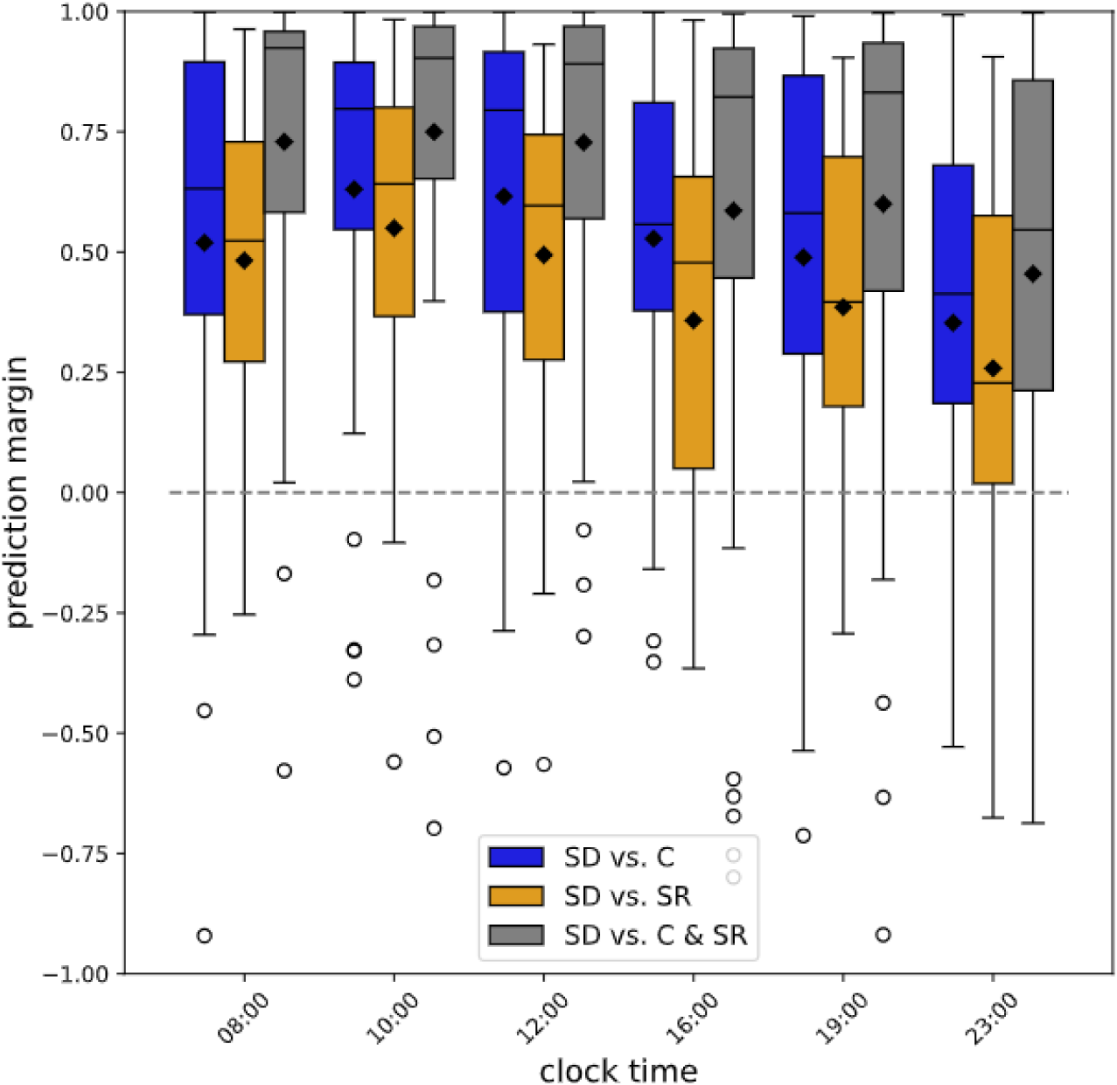
Model prediction performances across the different time points. The prediction margins were computed as the differences between each correct prediction probability and the corresponding probability toward the false prediction category. For example, if the sample belonged to the positive category (condition true = 1), the margin was calculated as P­(condition 1) subtracted by P­(condition 0); and vice versa for the negative category case. Therefore, for each prediction, the best possible prediction margin is 1, the worst is −1. A model that was solely based on random guessing would always predict P(0) = 0.5 and P(1) = 0.5, therefore, it would always have a prediction margin of 0, represented as the dashed gray line. Boxplot representation for the SD vs C model (based on 10 features and 204 predictions) in blue, the SD vs SR model (based on 10 features and 204 predictions) in orange, and the SD vs C & SR model (based on 12 features and 306 predictions) in gray. Box edges represent the interquartile range (IQR, Q1–Q3) with whiskers extended to 1.5× IQR. Data points outside the whisker bounds (outliers) are displayed as circles. Line inside the boxes represent the median values, diamonds the mean values.

### Sleep Dependency and Model Sanity

In the sleep dependency test, the SD vs C model (I), the SD vs SR model (II), and the overall SD model (III) correctly classified 88% (60 out of 68) samples, 81% (55 out of 68) samples, and 93% (95 out of 102) samples, respectively. The accuracies on the negative samples collected at t_1_ and t_8_ were better than the ones on the interventional samples collected between time points t_2_ and t_7_ (respective accuracies were 83%, 81%, and 91%, see Table 2). A quarter of all false classifications in this test were predictions on the same study subject, and the overall most misclassified time point (12 out of 28 false predictions) was t_8_ (at 08:00 in the morning after 8 h recovery sleep from 40 h sustained wakefulness). Although the rate of misclassifications was generally very low (12%, 28 out of 238), these findings highlight cases with considerable individual effects and that 8 h of sleep opportunity after a long wakefulness period may not be sufficient for few individuals to fully recover their metabolic traces of total sleep deprivation.

In the permutation tests, all three models performed no better or worse than random guessing when all class labels were randomly shuffled. The respective p-values were below 0.001 for all cases (see Figure S9, Text S7, and Table S8 for detailed results). These results provide strong evidence for the existence of a real and significant effect or association in the data. The robustness of the finding is highlighted by the fact that there is less than 0.1% chance of detecting such an extreme outcome if there was no real effect in the data. This underlines that our models have learned meaningful associations between the feature abundances and their corresponding label (i.e., the experimental conditions).

### Feature Inspection

Our findings so far demonstrate that sleep deprivation can be reliably detected using classification models based on 10 to 12 different molecular features in a single oral fluid sample. Each model’s feature shortlist with their molecular characteristics is presented in Text S8 and Tables S9–S11, as well as the abundance profiles across the different study interventions and classification tasks (Figures S10–S12). The shortlists of the one-vs-one classification models (I and II) have an overlap of three MFs. The shortlist of the overall SD classification model (III) contains MFs from both of the one-vs-one shortlists, with a majority overlapping with the SD vs SR model (II). However, three more yet lesser-ranked features appeared to be important for the overall SD classifier (ranks 6, 7, and 9). There is no general trend in the increase or decrease of molecular features indicating sleep deprivation, both directions occurred equally among the most important features (16 increases, 16 decreases). The mean odds ratios, which represent the mean weight of each feature in the classification task, range from 0.31 (weighed more than three times toward the negative label for decreasing features) to 3.33 (weighed more than three times toward the positive label for increasing features). Most of the shortlisted features were detected in HILIC+ mode, underlining the predominantly polar nature of salivary metabolites, in which is consistent with our pilot study.^36^

Integrating the results from the DLMO estimation, we constructed the feature abundance profiles in relation to circadian time (CT), with CT 0 representing the DLMO time of each sample donor in the evening before the respective intervention night. These profiles are visualized in Figure S13. Cosinor analysis of the DLMO-adjusted profiles revealed that 3 out of 10 features (SD vs C, model I), 4 out of 10 features (SD vs SR, model II), and 7 out of 12 features (overall SD model III) exhibited significant 24-h period rhythmicity, respectively. Detailed evaluations can be found in Text S9 and Tables S12–S14. These findings of feature rhythmicity and time-of-day variation are corroborating with other time-course studies in oral fluid.^17,36,60–62^ As a consequence, some features are discriminative in the morning hours, while others become important in the evening. In addition, wakefulness and sleep are regulated by the endogenous circadian clock. It is therefore pivotal to consider the individual circadian phase when searching for metabolic biomarkers of sleep loss.^17^ As demonstrated in several works, the metabolome incorporates the circadian phase information.^63–66^ Therefore, those features that follow a nearly 24-h period rhythm as a function of the individual DLMO time serve as circadian phase indicators for our models for the appropriate use of each discriminative feature. The implementation of more rhythmic features may also contribute to the superior performance of the overall SD model (7 out of 12 features) compared to the one-vs-one classification models (3 and 4 rhythmic features out of 10 features, respectively). However, our study design cannot show whether these metabolites follow a diurnal or truly circadian rhythm, as we opted for a realistic study environment rather than a constant routine protocol that would eliminate all external factors to assess true circadian rhythmicity.^67^

### Legal Implications and Limitations

Due to the absence of reliable biomarkers, some jurisdictions have adopted practical legal standards. For instance, New Jersey’s Maggie’s Law allows the prosecution of drivers who have been awake for more than 24 consecutive hours at the time of a fatal accident, classifying such behavior as reckless driving.^68^ Moreover, an expert committee found a consensus that “drivers who have slept for 2 h or less in the preceding 24 h are not fit to operate a motor vehicle”.^69^ As a result, the 24-h wakefulness threshold has gained forensic significance and was represented in the sleep deprivation (SD) intervention of this study. Our results demonstrate the feasibility of building reference-free metabolomics-based sleep loss detection models according to Maggie’s Law. Importantly, the models presented here are investigational and designed to assess theoretical potential rather than provide immediate practical application. In this regard, we note that our models worked well for healthy men adhering to a regular day-night schedule. As a next step, the shortlisted molecular features should be identified and tested for further robustness in females and individuals who are under the influence of possible confounders, such as shiftwork, comorbidities, or drug and stimulant use. Therefore, although this study enrolled more participants than all previous studies with comparable protocols and aims,^17,19,21,23^ the relevant features should be tested in larger and more diverse cohorts before practical application. In addition, it would be rewarding to see which exact amount of sleep loss is required to induce the metabolic fingerprint of sleep deprivation, similar to a dose–response relationship analysis. Our study has demonstrated that repeated sleep restriction to 6 h per night in habitual 8-h sleepers was insufficient in this regard. Lastly, it should be emphasized that the present models are designed to identify sleep-deprived rather than sleep-impaired states. Although these conditions can overlap, individual susceptibility to sleep-related impairments needs to be considered in specific use cases. Nevertheless, our work can make a significant contribution to the overarching goal, i.e., the metabolic fingerprint of sleep loss-induced impairment.

## Conclusions

This study was designed to explore the theoretical potential and limitations of reference-free metabolomics-based sleep loss detection in a realistic setting with an emphasis on forensic applications using oral fluid.

The results hold promise for detecting sleep-deprived individuals based on their metabolic fingerprint in oral fluid. By contrast, repeated moderate sleep restriction did not lead to exploitable metabolic changes when compared with controlled sleep. Owing to the complex nature of endogenous metabolism, machine learning models aid in precise classification of unseen samples without the need for an additional reference sample from the same individual, making them suitable for forensic applications. These models should be based on a panel of molecular features that also contain information about circadian phase.

Further validation in larger heterogeneous cohorts under common confounding factors and unambiguous identification of relevant metabolites are warranted for routine applications such as roadside or point-of-care testing, as well as for the understanding of biological mechanisms driving these observations.

## Supplementary Material



## Data Availability

The data supporting the findings of this study are available within the paper and its Supporting Information. Raw MS data files were uploaded to the MetaboLights study identifier MTBLS11413, accessible via www.ebi.ac.uk/metabolights/MTBLS11413. All code was made publicly available on https://gitlab.uzh.ch/zifm-fpt/ME-SMART.

## Notes

Figure S1: consort diagram; Text S1: oral fluid collection; Figure S2: sleep regimes and oral fluid sampling protocol; Text S2: DLMO estimation; Table S1: LC instrument setup (targeted); Table S2: MS instrument setup (targeted); Table S3: MRM settings; Text S3: metabolic fingerprinting; Table S4: LC instrument setup (untargeted); Table S5: MS instrument setup (untargeted); Table S6: MS-DIAL software settings; Text S4: data preprocessing; Figure S3: machine learning model loop; Text S5: classification model training; Table S7: ML hyperparameters; Figure S4: ML hyperparameter tuning; Equation S1: F_0.5_ score calculation; Figure S5: DLMO times; Figure S6: ML model results (SD vs C); Figure S7: ML model results (SD vs SR) Figure S8: ML model results (SD vs C and SR); Text S6: ML model evaluations; Text S7: ML model sanity; Figure S9: permutation test results; Table S8: permutation test result values; Text S8: feature inspection; Table S9: most important features (SD vs C); Table S10: most important features (SD vs SR); Table S11: most important features (SD vs C and SR); Figure S10: daily profiles of SD vs C features; Figure S11: daily profiles of SD vs SR features; Figure S12: daily profiles of SD vs C and SR features; Text S9: cosinor analysis; Table S12: cosinor analysis results (SD vs C); Table S13: cosinor analysis results (SD vs SR); Table S14: cosinor analysis results (SD vs C and SR); Figure S13: cosinor model fittings; Text S10: chemicals, reagents, and materials (PDF)
