# Supplementary material for: Leveraging the Metabolic Fingerprint of Sleep Deprivation and Sleep Restriction for Forensic Applications: A Machine Learning Study in Oral Fluid Metabolomics
Source: J Proteome Res. 2026 May 6;25(6):2740–50. doi: 10.1021/acs.jproteome.5c01064 (PMC13247977; doi:10.1021/acs.jproteome.5c01064)
Supplement: Supplementary file 1 [file pr5c01064_si_001.pdf]

Supporting Information

Leveraging the Metabolic Fingerprint of Sleep Deprivation and Sleep Restriction for Forensic Applications: A Machine Learning Study in Oral Fluid Metabolomics

*Michael Scholz[1], Andrea E. Steuer[1], Akos Dobay[2], Hans-Peter Landolt[3,4+], Thomas Kraemer[1,4+]**

[1] Department of Forensic Pharmacology and Toxicology, Zurich Institute of Forensic Medicine, University of Zurich, Zurich, Switzerland.

[2] Forensic Machine Learning Technology Center, University of Zurich, Zurich, Switzerland.

[3] Institute of Pharmacology and Toxicology, University of Zurich, Zurich, Switzerland.

[4] Sleep & Health Zurich, University of Zurich, Zurich, Switzerland.

*corresponding author, thomas.kraemer@irm.uzh.ch

[+]shared last authorship

Table of Contents

List of each Supporting Material (in Order of Appearance) 3
Additional Experimental Section 4
Study Protocol and Cohort 4
Oral Fluid Collection 5
Dim-Light Melatonin Onset Estimation 6
Metabolic Fingerprinting 7
Classification Model Training 9
Model Evaluations and Optimization for Forensic Applications 11
Additional Results 12
DLMO estimation 12
Model Evaluations and Optimization for Forensic Applications 13
Sleep Dependency and Model Sanity 17
Feature Inspection 19
Additional Information 27
Chemicals, Reagents, and Materials 27
Abbreviations 28
References 29

List of each Supporting Material (in Order of Appearance)

Figure S1.	Consort diagram
Text S1.	Oral fluid collection
Figure S2.	Sleep regimes and oral fluid sampling protocol
Text S2.	DLMO estimation
Table S1.	LC instrument setup (targeted)
Table S2.	MS instrument setup (targeted)
Table S3.	MRM settings
Text S3.	Metabolic fingerprinting
Table S4.	LC instrument setup (untargeted)
Table S5.	MS instrument setup (untargeted)
Table S6.	MS-DIAL software settings
Text S4.	Data pre-processing
Figure S3.	Machine learning model loop
Text S5.	Classification model training
Table S7.	ML hyperparameters
Figure S4.	ML hyperparameter tuning
Equation S1.	$F_{0.5}$ score calculation
Figure S5.	DLMO times
Figure S6.	ML model results (SD vs. C)
Figure S7.	ML model results (SD vs. SR)
Figure S8.	ML model results (SD vs. C and SR)
Text S6.	ML model evaluations
Text S7.	ML model sanity
Figure S9.	Permutation test results
Table S8.	Permutation test result values
Text S8.	Feature inspection
Table S9.	Most important features (SD vs. C)
Table S10.	Most important features (SD vs. SR)
Table S11.	Most important features (SD vs. C and SR)
Figure S10.	Daily profiles of SD vs. C features
Figure S11.	Daily profiles of SD vs. SR features
Figure S12.	Daily profiles of SD vs. C and SR features
Text S9.	Cosinor analysis
Table S12.	Cosinor analysis results (SD vs. C)
Table S13.	Cosinor analysis results (SD vs. SR)
Table S14.	Cosinor analysis results (SD vs. C and SR)
Figure S13.	Cosinor model fittings
Text S10.	Chemicals, reagents, and materials

Additional Experimental Section

Study Protocol and Cohort

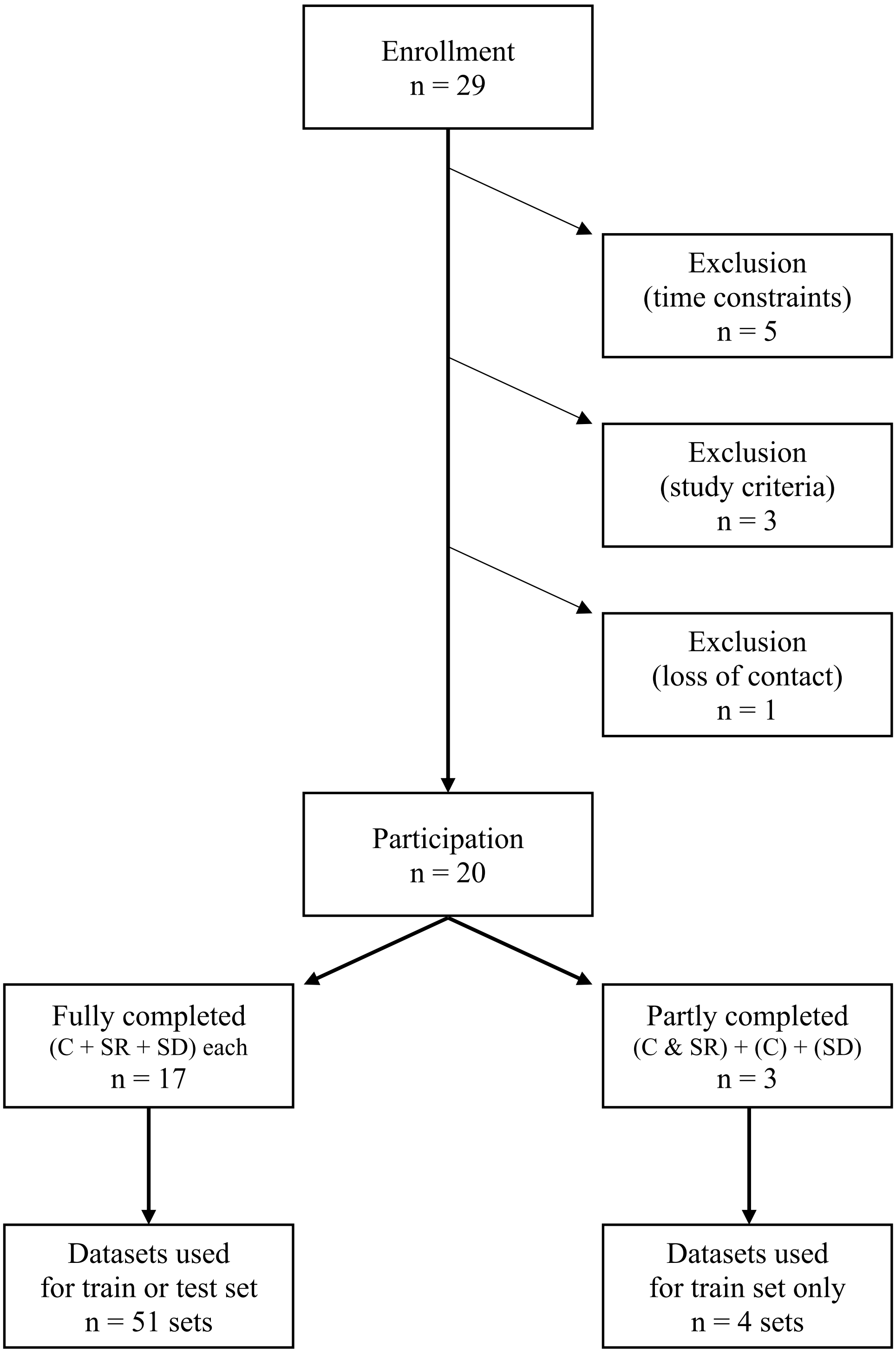

Figure S1: Consort diagram

Oral Fluid Collection

Text S1. Given the sampling protocol (see Figure S2), it is meaningful to examine the changes that occur following the intervention night (after time point t_1), specifically at time points t_2, t_3, t_4, t_5, t_6, and t_7. The results from the samples taken at time point t_8 (after the recovery night) serve as validation of the sleep-wake dependency of the metabolites. The fundamental reason is that changes in metabolism are only caused by extended periods of wakefulness if they return to their original state after a period of recovery sleep (i.e., an adaptive homeostatic response) – if the recovery period is long enough and the recovery process is sufficiently fast.[1] In studies on performance and working memory, a recovery sleep period of 8 hours at scheduled nighttime was sufficient to attenuate acute sleep deprivation effects to near baseline.[2,3] Hence, if there is a difference in metabolite levels between the study conditions determined after the recovery night (t_8), they should not be considered as being associated with adaptive sleep-wake regulation and should be removed from further analysis. A similar concept is applied for time point t_1, as there is no anticipated statistical difference between the sleep deprivation and the control condition at this time point since they share the same sleep-wake protocol until this stage. Thus, the exclusion of molecular features that are non-compliant with these concepts further strengthens the validity of the results and the underlying hypothesis.
Salivette® cotton swabs (No. 51.1534) were used for metabolomics samples, Salivette® synthetic swabs (No. 51.1534.500) were used for melatonin samples.

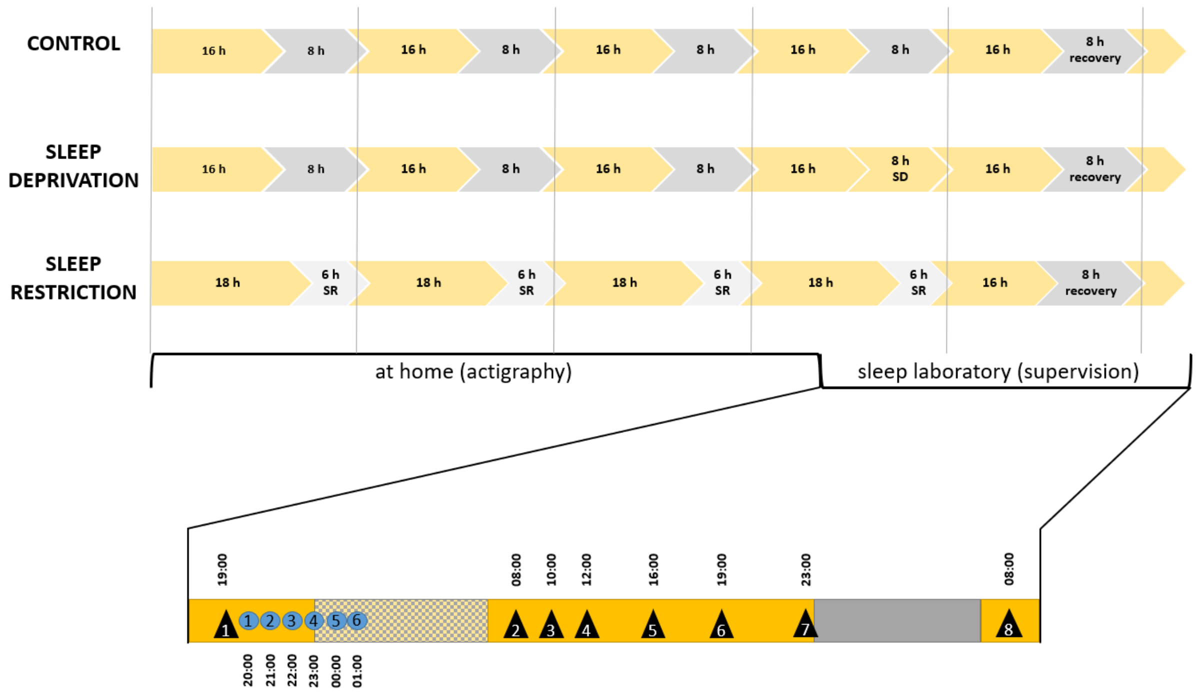

Figure S2: Sleep regimes and oral fluid sampling protocol
Top: Overview of sleep regime in different study arms (control, sleep deprivation, sleep restriction). Yellow colors indicate time awake, gray colors bed times, vertical lines a period of 24 h. Abbreviations: SD, sleep deprivation; SR, sleep restriction.
Bottom: Oral fluid sampling protocol. Yellow background represents scheduled wake times, grey background scheduled sleep time (recovery night). Yellow background with grey structure indicates intervention night: Scheduled sleep (23:00 – 07:00 for control condition, 01:00 – 07:00 for sleep restriction condition), scheduled wake for sleep deprivation condition. Black triangles indicate collection time points of oral fluid for untargeted metabolomics analysis, clock times above. Blue circles indicate collection time points of oral fluid for targeted melatonin analysis, clock times below. Melatonin collection time points 5 and 6 were only carried out in sleep restriction and sleep deprivation conditions.

Dim-Light Melatonin Onset Estimation

Text S2. For this analysis, the thawed sample aliquots were added with 25 µL of internal standard solution (melatonin-d_4 in methanol at 1.2 ng/mL) and transferred into the Biotage® Extrahera (Biotage, Uppsala, Sweden). Herein, samples were extracted using Isolute SLE+ columns (absorption time 5 min, two elution cycles with 1.5 mL ethyl acetate with an intermediate wait time of 7 min). A volume of 5 µL DMSO was added to the extracts before drying under gentle nitrogen stream in a Turbovap® (Biotage, Uppsala, Sweden) and resuspension in 40 µL methanol and 40 µL reconstitution solution (0.2% V/V ammonium formate in water). A blind sample was prepared analogously. Quantification of 20 µL of prepared extract was performed by LC-MS/MS analysis (Phenomenex® Kinetex® F5 chromatography column, 100 mm x 2.1 mm i.d., 2.6 µm particle size in a Prominence UFLC system from Shimadzu, Kyoto, Japan coupled to QTRAP® 6500+ linear ion trap quadrupole mass spectrometer in positive ESI mode from Sciex, Concord, Ontario, Canada). The setup and all settings are presented in Tables S1-S3. Controlled by Analyst software 1.7.3 (Sciex), the following transitions were used: 232.9→174.0 Da (melatonin quantifier), 232.9→130.0 Da (melatonin qualifier), 237.2→178.0 Da (melatonin-d_4 quantifier), 237.2→134.2 Da (melatonin-d_4 qualifier).

Raw signals from melatonin quantification analysis were extracted, integrated, and converted into concentrations by Skyline software for small molecules (version 22.2.0.527).[4]

Table S1: LC instrument setup of the Shimadzu Prominence UFLC system

Mobile Phase A	0.1% (v/v) 1M ammonium formate, 0.1 % (v/v) formic acid in water
Mobile Phase B	0.1% (v/v) 1M ammonium formate, 0.1 % (v/v) formic acid in acetonitrile
Flow rate	0.6 mL/min
Flow gradient	0 – 0.5 minute 97% A;<br>0.5 – 4 minutes gradual increase to 40% B;<br>4 – 5 minutes gradual increase to 95% B;<br>5 – 6 minutes held at 95% B and then decreased to start conditions and<br>re-equilibration for 1 minute
Column temperature	40°C
Injection volume	20 µL

Table S2: MS instrument setup of Sciex QTRAP® 6500+ (linear ion trap quadrupole)

Source type	ESI
Source temperature	450°C
Curtain gas	45 psi
Ion source gas 1	70 psi
Ion source gas 2	60 psi
Ion-spray voltage floating	5500 V
Declustering potential	46 eV
Entrance potential	10 eV
Polarity	positive
Resolution	unit (Q1 and Q3)

Table S3: Multiple reaction monitoring (MRM) settings

ID	Q1 mass (Da)	Q3 mass (Da)	Dwell time (msec)	Collision energy (eV)	Collision cell exit potential (eV)
Melatonin 1 (quantifier)	232.9	174.0	100	19	10
Melatonin 2 (qualifier)	232.9	130.0	100	61	16
Melatonin-*d*4 1 (quantifier)	237.2	178.0	100	19	10
Melatonin-*d*4 2 (qualifier)	237.2	134.2	100	57	8

Metabolic Fingerprinting

Text S3. For the untargeted metabolomics analysis, a volume of 900 µL extraction solution composed of acetonitrile/acetone/methanol (8/1/1, V/V/V) was added to the thawed sample aliquots, vortex mixed, shaken for 10 minutes at 1400 rpm (ThermoMixer, Vaudaux-Eppendorf AG, Schönenbuch, Switzerland), and incubated overnight at -20°C. On the following day, the mixture was thawed and centrifuged (5 min at 20'000 *g*) before 200 µL of the supernatant was transferred to conical glass vials. A blind sample (using 2 mL of water instead of oral fluid in the Salivette® device, i.e., a process blank sample) was prepared accordingly to correct for false positive process results. Additionally, a pooled quality control (QC) sample was prepared by combining 10 µL of each oral fluid sample, divided into numerous aliquots, and processed in the same manner as described above. Lastly, a dilution series of pooled QC samples (100%, 80%, 60%, 40%, and 20% concentrations) was made by diluting with the extraction solution. The prepared sample extracts were then analyzed in batches via liquid chromatography coupled to high-resolution mass spectrometry (LC-HRMS) as described in detail elsewhere.[5] In brief, we separately used both a reversed-phase (RP) chromatography column (Waters XSelect HSST RP-C18 column, 150 mm x 2.1 mm i.d., 2.5 µm particle size) and a hydrophilic interaction chromatography (HILIC) column (Merck SeQuant ZIC HILIC, 150 mm x 2.1 mm i.d., 3.5 µm particle size) on a Thermo Fischer UltiMate 3000 UHPLC system (Thermo Fischer Scientific, San Jose, CA, USA). The LC system was connected to a high-resolution quadrupole-time-of-flight instrument system (TripleTOF 6600, Sciex, Concord, Ontario, Canada), equipped with an electrospray ionization (ESI) source applying either positive or negative ionization. Hence, all samples were measured in four different LC-MS modes (in the manuscript referred to as RP+, RP-, HILIC+, and HILIC-) to cover the broad chemical diversity of metabolites in oral fluid. Tables S4-S5 offer detailed chromatography gradient profiles and MS instrument settings. Analyst software 1.7 (Sciex) controlled the generation of tandem mass spectra by data-dependent acquisition (DDA, top 5). System suitability was assessed by retention time and peak intensity of a model metabolite mix (arginine, cortisol, cortisone, creatinine, glycocholic acid, hippuric acid, leucine, raffinose, riboflavin, tryptophan) before each run.[6] Each sample sequence started with the injection of three pooled QC samples for equilibration, a blind QC sample, the ascending dilution series of pooled QC samples, and two further pooled QC samples, before blocks of nine randomized study samples and a pooled QC sample were injected repeatedly. Each recorded pooled QC sample was used for online monitoring of instrument performance. Recalibration of MS1 and MS2 signals was executed automatically after every 10 samples by the system software. If any of these QC specifications was not met, the system was stopped, and the analytical batch rerun after successful troubleshooting.

Table S4: LC instrument setup of Thermo Fischer UltiMate 3000 UHPLC system

Mobile Phase A	10mM ammonium formate, 0.1% (v/v) formic acid in water
Mobile Phase B	0.1% (v/v) formic acid in methanol
Mobile Phase C	25mM ammonium acetate, 0.1% (v/v) acetic acid in water
Mobile Phase D	0.1% (v/v) acetic acid in acetonitrile
Flow rate	0.5 mL/min
RP gradient	0-1 min: 100% A;<br>1-15 min: gradual increase to 100% B;<br>15-18 min: held at 100% B for 3 minutes; then decreased to start conditions with flow rate to 0.7 mL/min and re-equilibration for 2 minutes
HILIC gradient	0-1 min: 5% C / 95% D;<br>1-10 min: gradual change to 60% C / 40% D;<br>10-12 min: gradual change to 90% C / 10% D;<br>12-13 min: hold conditions for 1 minute; then abrupt change to start conditions of 5% C / 95% D and re-equilibration for 2 minutes
Column temperature	40°C
Injection volume	5 µL

Table S5: MS instrument setup of Sciex TripleTOF 6600 (QTOF)

Source type	ESI
Source temperature	450°C
Curtain gas	25 psi
Ion source gas 1	50 psi
Ion source gas 2	60 psi
Ion-spray voltage floating	5500 V
Declustering potential	80 eV
Mass range	m/z 50 to m/z 1000
Accumulation time	50 msec
Collision energy	35 eV
Collision energy spread	15 eV
DDA settings	top 5 MS2, dynamic background subtraction on the five most intense ions; intensity threshold above 100 counts per second (cps); exclusion time of 5 sec (half peak width) after two occurrences

Table S6: MS-DIAL software settings for the respective LC-MS mode raw data files

Parameter	HILIC +	HILIC -	RP +	RP -
Retention time begin	0.5	0.5	0.5	0.5
Retention time end	15	15	20	20
Mass range begin	50	50	50	50
Mass range end	1000	1000	1000	1000
MS2 mass range begin	50	50	50	50
MS2 mass range end	1000	1000	1000	1000
MS1 tolerance	0.04	0.04	0.04	0.05
MS2 tolerance	0.025	0.025	0.025	0.025
Maximum charged number	2	2	2	2
Smoothing method	Linear Weighted Moving Average			
Smoothing level	4	4	4	4
Minimum peak width	5	5	5	5
Minimum peak height	500	1000	400	150
Mass slice width	0.05	0.04	0.04	0.05
Sigma window value	0.5	0.5	0.5	0.5
MS2Dec amplitude cut off	0	0	0	0
Exclude after precursor	True	True	True	True
Keep isotope until	0.5	0.5	0.5	0.5
Keep original precursor isotopes	False	False	False	False
Adduct ions	[M+H]+<br>[M+NH4]+<br>[M+Na]+<br>[M+ACN+H]+<br>[M+H-H2O]+<br>[M+H-2H2O]+	[M-H]-<br>[M-H2O-H]-<br>[M+Hac-H]-	[M+H]+<br>[M+NH4]+<br>[M+Na]+<br>[M+CH3OH+H]+<br>[M+H-H2O]+<br>[M+H-2H2O]+	[M-H]-<br>[M-H2O-H]-<br>[M+FA-H]-
Gap filling by compulsion	True	True	True	True

Text S4. Zero values, resulting from MS-DIAL processing for a molecular feature (MF) that was neither detected nor gap-filled in a sample, were converted into missing values (i.e., NaN values). Then, we applied batch and run-order correction (using nPYc-Toolbox package[7], version 1.2.8) with reference to pooled QC samples (adapted LOWESS approach proposed by Dunn et al.[8]), parameter batch_correction_window=7). Next, MF were filtered by several measurement robustness criteria concerning their characteristics in pooled QC samples.[9] Any MF was kept if all of the following requirements were met: a relative standard deviation of less than 20%, a correlation to the dilution series (Pearson coefficient) of more than 0.5, a fold-change to the blind sample of more than 3 for RP data, and more than 4 for HILIC data, respectively, and a detection rate in pools of more than 70%.[8–12] Probabilistic quotient normalization was then applied to account for technical variance and different dilution of samples, based on their stable metabolic content.[13] Peak areas were further log-transformed (base 10) and auto-scaled (i.e., z-score normalized). The "80% rule" was applied so that a MF was kept if it contained less than 20% missing values.[14] The remaining missing values (less than 4% of all values) were imputed by applying k-nearest neighbor method (KNNImputer module from scikit-learn[15] version 1.5.0, parameters: n_neighbors=10, weights='distance').[16,17] Lastly, the four datasets were combined while deleting duplicate features, e.g., a metabolite that is detected in two or more LC-MS conditions is only kept once where most abundant.

Classification Model Training

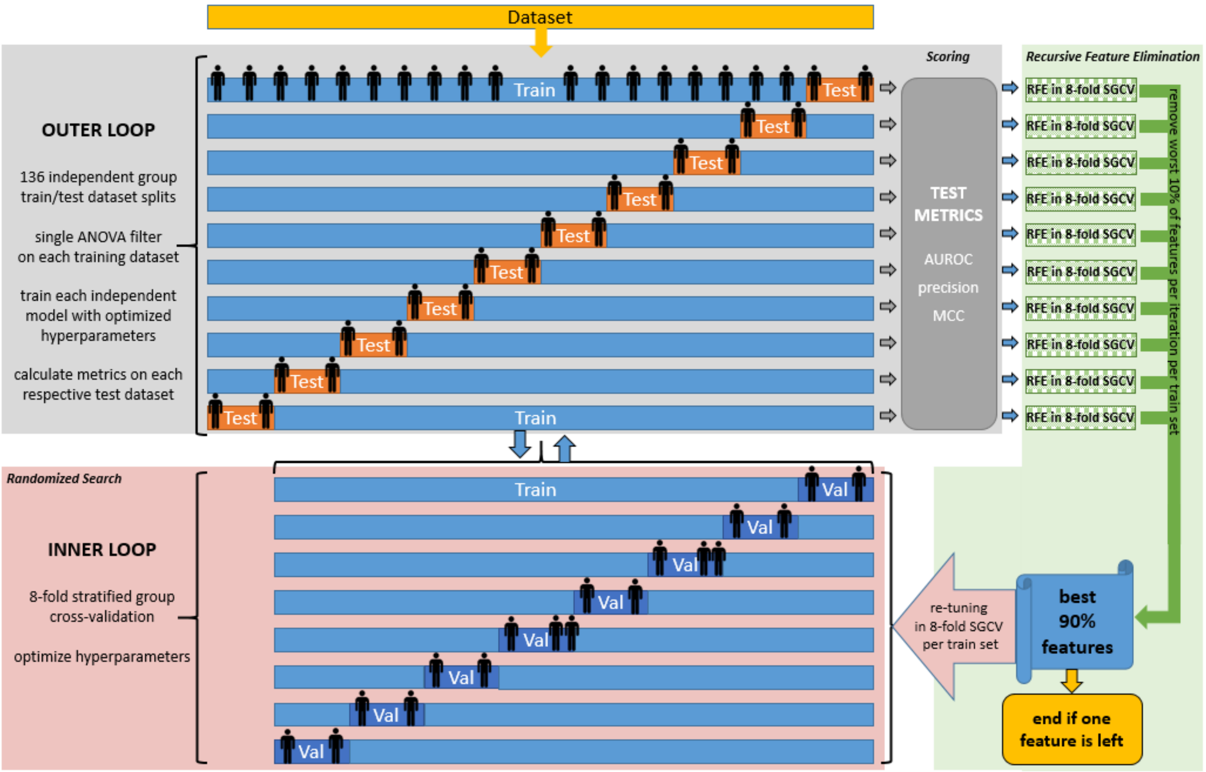

Figure S3: Nested cross-validation and recursive feature elimination loop
For each independent dataset split, the training data is used for model hyperparameter optimization (inner loop, red zone) by splitting into separate training and validation parts (represented in different shades of blue). The optimized model is then trained on the respective training data (blue data) and scored on the unseen corresponding test data (orange data) in the outer loop (grey zone). Then, recursive feature elimination is applied to remove the least important 10% of features (green zone). The remaining 90% of features enter the inner loop again as the new training dataset. This loop continues until one feature is left in the training dataset. Abbreviations: ANOVA: analysis of variance, AUROC: area under the receiver operator characteristic curve, MCC: Matthews correlation coefficient, RFE: recursive feature elimination, SGCV: stratified group cross-validation.

Text S5. As illustrated in Figure S3, the loop consists of three repeated steps: Firstly, the hyperparameters of the respective algorithm are optimized on the training dataset in a randomized grid search under 8-fold cross-validation (scikit-learn module RandomizedSearchCV). Secondly, the optimized model was fitted on the training dataset and its generalization abilities were evaluated by predicting the unseen data from the test dataset. Lastly, for the sake of interpretability in a biological context, recursive feature elimination (RFE) with 8-fold cross-validation (scikit-learn module RFECV) was applied to the fitted model to select the most important features responsible for classification power. In this analysis, one or more features (depending on the size of the parameter 'step') of the training dataset are removed during each iteration, and the decrease of the model's classification score is evaluated by a k-fold cross-validation procedure (here, 8-fold cross-validation). The features causing the lowest drop in the score (i.e., the features deemed the most unimportant) are eliminated first. This continues until the desired number of (important) features is left (parameter 'min_features_to_select'). Depending on the least acceptable classification score, one can thus shrink the feature table until a minimum set of features is left that can still discriminate with acceptable results, hereby simplifying the model and its application in a broader context. For optimal use of computational resources, we shrunk the number of features by 10% during each loop (i.e., the worst 10% of features were dropped per loop), and linearly increased the number of iterations for hyperparameter combinations in the randomized grid search per loop (see Figure S4). A list of the allowed hyperparameter ranges and the iteration gradient are presented in Table S7. The chances of finding the optimal hyperparameter combination increases with the number of tries (iterations) but trades off versus run time, especially for high-dimensional datasets. Because not all hyperparameters are equally important, the randomized search with a sensible number of iterations is able to find models that perform as good as or often better than models found with a manual grid search, while saving a considerable amount of computational resources and time.[18] Model hyperparameter were

optimized with respect to maximizing the area under the receiver operator characteristic curve (AUROC), the precision score, and the Matthews correlation coefficient (MCC) in this order of priority. As mentioned above, the model training, its hyperparameter optimization, and its evaluations on the unseen test data were performed 136 separate times on all possible dataset splits in a nested cross-validation procedure, and each classification metric's mean were reported.

Table S7: Hyperparameters of LogisticRegression (the logistic regression classifier) used for the randomized search

Parameter	Setting	Value
solver	set	saga
penalty	set	elastic net
L1 penalty ratio	optimized	uniform distribution 0-1
C	optimized	log-uniform distribution 10^{-4}-1
class weight*	optimized	None or balanced
tolerance	set	0.001
maximum iterations	set	2000
random state	set	101010101

**The hyperparameter "class weight" only appears in one-vs-rest classification (SD vs. C and SR).*

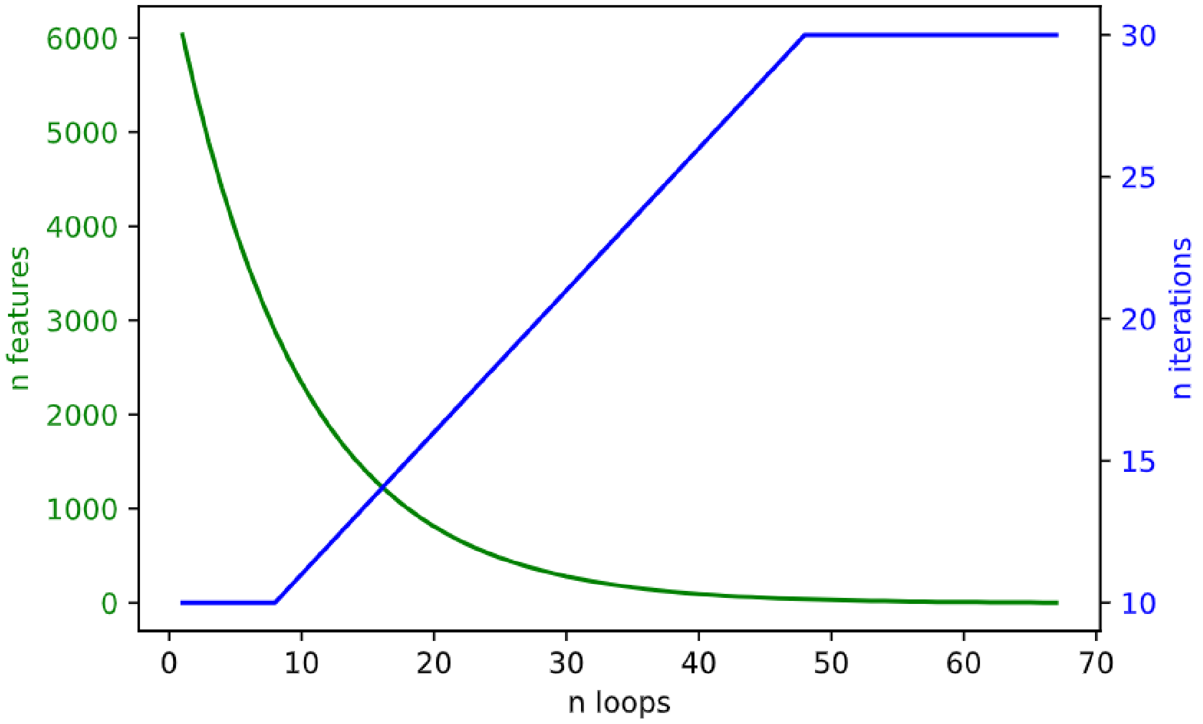

Figure S4: Gradient increase of iterations in randomized hyperparameter search with respect to decreasing amount of total features during recursive feature elimination loops
As the number of features decreases during recursive feature elimination (green line, y-axis to the left) per loop (x-axis), the number of randomized hyperparameter tries increases (blue line, y-axis to the right). The number of iterations is set to 10 in the beginning. As the number of features comes below 3000, the iterations increase by 0.5 per loop until they reach the preset limit of 30 iterations per loop. For the additional hyperparameter in the one-vs-rest classification, the number of iterations starts with 15 and is limited to 50 (not shown in the graph).

Model Evaluations and Optimization for Forensic Applications

Equation S1: $F_{0.5}$ score calculation. The $F_{0.5}$ score can be calculated from the number of true positives (tp), false positives (fp), and false negatives (fn); and its deduction from the precision and recall scores.

$$F_{0.5} = \frac{1.25 \times tp}{1.25 \times tp + fp + 0.25 \times fn} = 1.25 \left(\frac{precision \times recall}{0.25 \times precision + recall}\right)$$

Additional Results

DLMO estimation

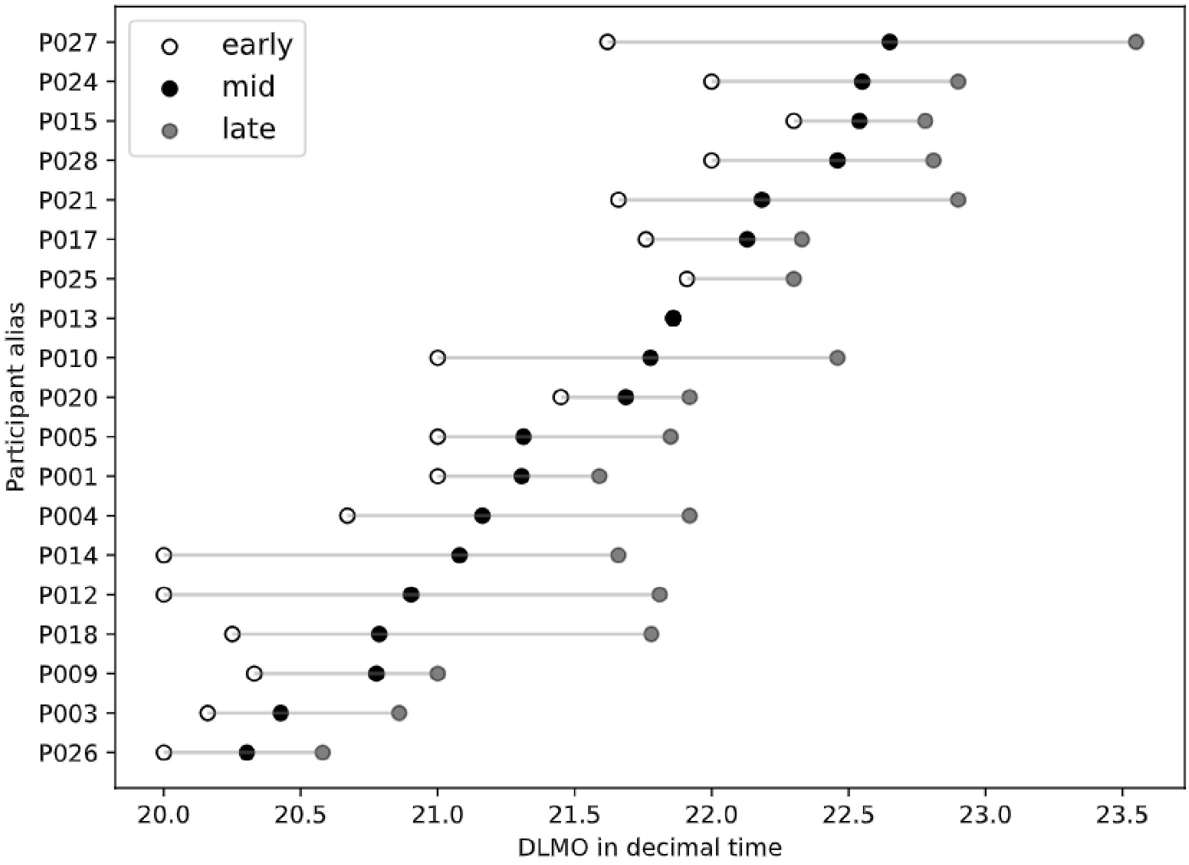

Figure S5: Dim-light melatonin onset (DLMO) times
During each visit, each participant's DLMO evening profile was constructed and the DLMO time was computed using the hockey-stick method (see Methods). 54 out of 55 profiles could be interpreted (all except the only profile of P016, therefore not listed).

Model Evaluations and Optimization for Forensic Applications

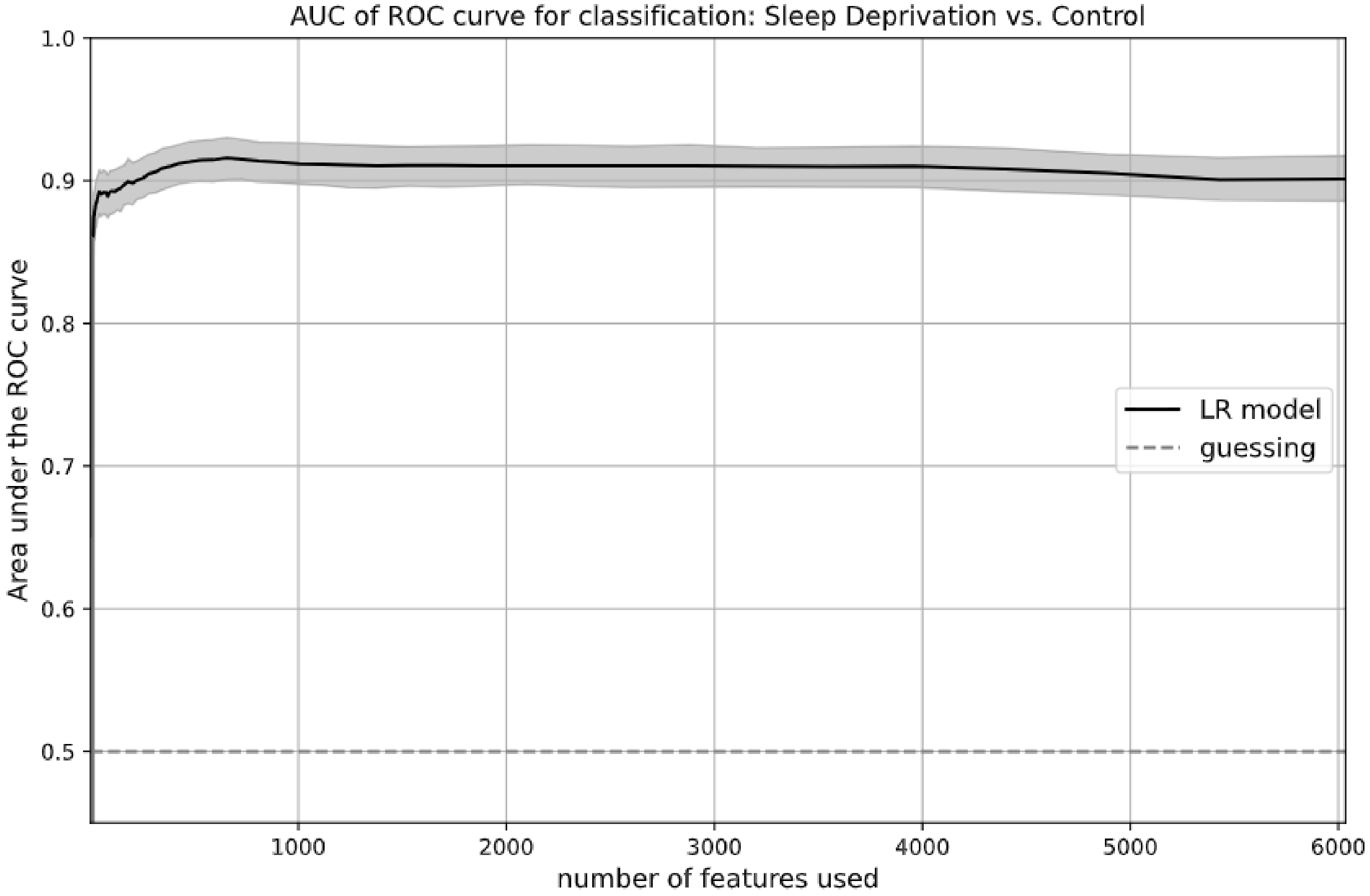

Figure S6: Graphical results of the looped randomized hyperparameter search with recursive feature elimination for sleep deprivation (SD) vs. control (C) intervention
Visualization of area under the receiver operator characteristic curve (AUROC) against number of features used. Line with shade represents logistic regression (LR) model mean with 95% confidence interval. Dashed line at AUROC = 0.5 represents random guessing model.

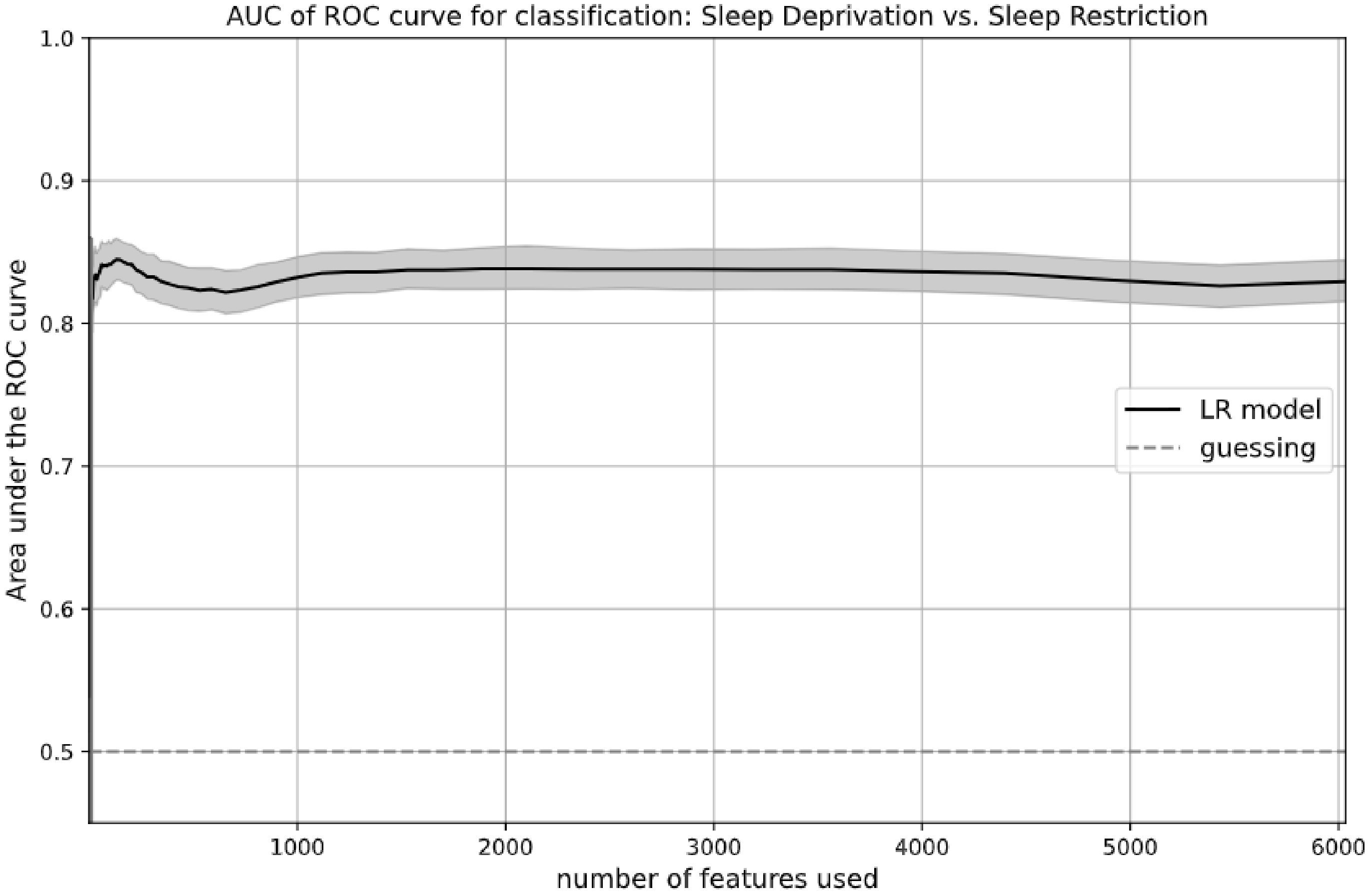

Figure S7: Graphical results of the looped randomized hyperparameter search with recursive feature elimination for sleep deprivation (SD) vs. sleep restriction (SR) intervention
Visualization of area under the receiver operator characteristic curve (AUROC) against number of features used. Line with shade represents logistic regression (LR) model mean with 95% confidence interval. Dashed line at AUROC = 0.5 represents random guessing model.

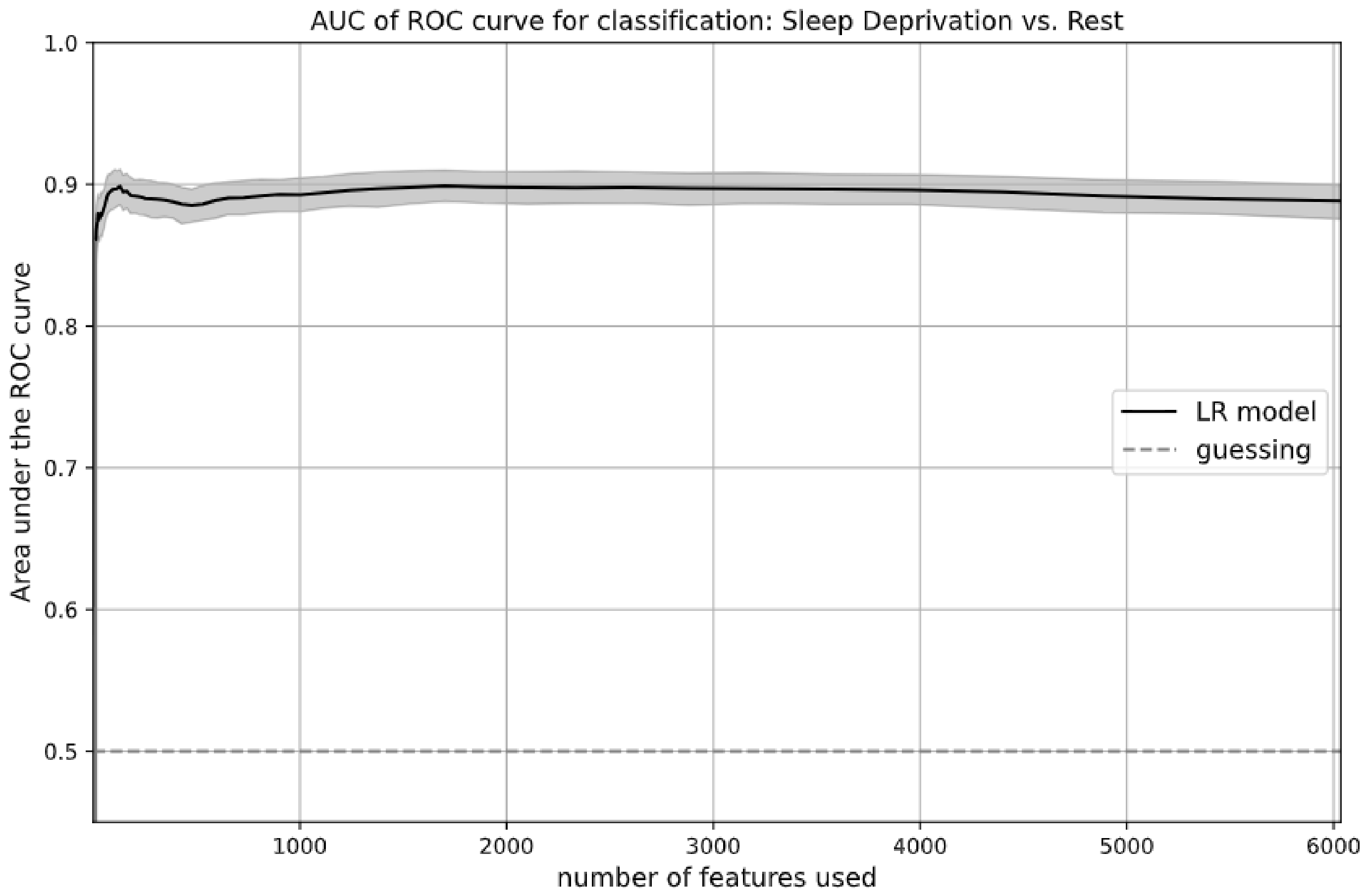

Figure S8: Graphical results of the looped randomized hyperparameter search with recursive feature elimination for sleep deprivation vs. control and sleep restriction intervention classification
Visualization of area under the receiver operator characteristic curve (AUROC) against number of features used. Line with shade represents logistic regression (LR) model mean with 95% confidence interval. Dashed line at AUROC = 0.5 represents random guessing model.

Text S6. The area under the ROC curve (AUROC) is an established indicator of machine learning (ML) model performance. It can clearly be seen that the predictive LR models perform better than random guessing (dotted line of AUROC = 0.5) which is the comparative analysis (as there are currently no valid biomarkers for sleep deprivation in oral fluid, the status quo is equal to random guessing). In addition, it is shown that there exists a distinct number of features after which the inclusion of more features does not add significant value to the model's AUROC, i.e., there is an optimal number of features for the given models.
These results follow a certain logic of ML, also known as the curse of dimensionality:[19] A model with a single feature input often has poor classification power (low AUROC for n features = 1). Consequently, including some more meaningful features allows the model to perform better (AUROC increasing for low feature number) until a peak is reached (maximum of AUROC curve). With more features and thus more noise and dimensions being added to the model, its power does not improve anymore but gradually decreases (gradual decrease of AUROC for high feature numbers).

Sleep Dependency and Model Sanity

Text S7. In this test, all dataset labels (C, SD, or SR) are randomly shuffled before model training, and then the test samples are classified. This prediction should yield poor scores (e.g., trained model accuracy is not superior to guessing or a coin flip) as the new labels are meaningless, which would underline a real biological effect in the original dataset. If the model still has strong performance on both datasets with shuffled labels, it indicates that the model is likely overfitting or capturing irrelevant information instead of useful patterns.[20] A classifier that predicts by random guessing would yield a $F_{0.5}$ score of 0.5 in the case of a balanced class distribution (valid for SD vs. C, and SD vs. SR). In the SD vs. C & SR case, the class imbalance is 1/3 SD (positive) cases to 2/3 C & SR (negative) cases. Therefore, a random guessing classifier would yield a precision of 1/3 and a recall of 0.5, which leads to a $F_{0.5}$ score of approximately 0.357.

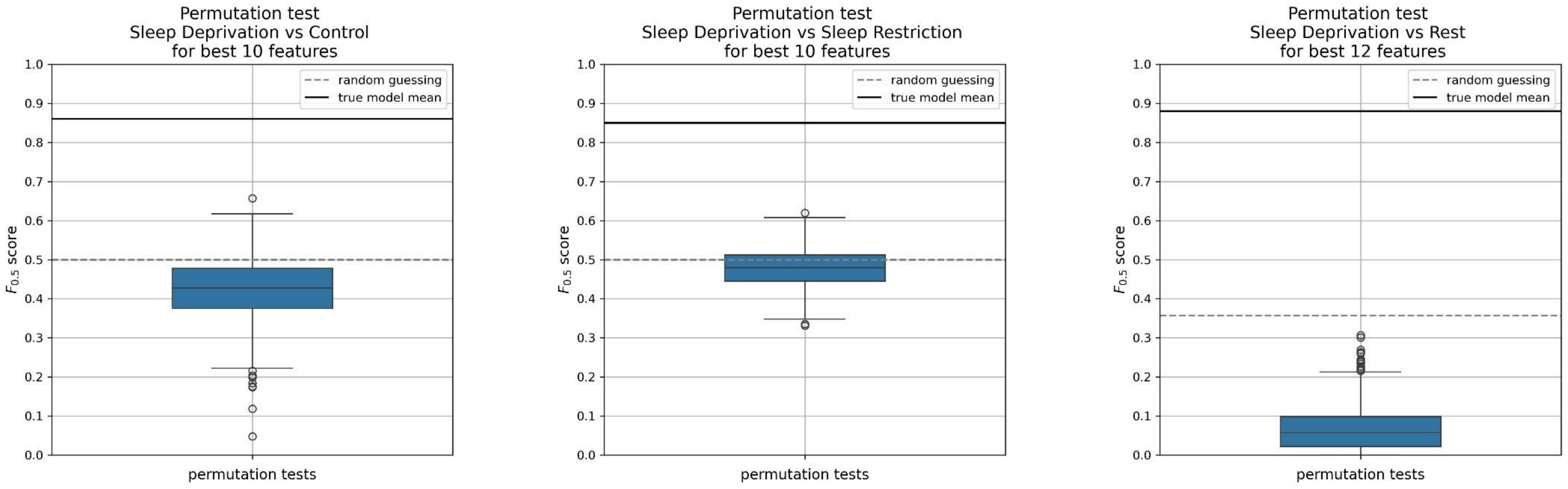

Figure S9: Permutation test results
The results of the SD vs. C classifier (left), the SD vs. SR classifier (center), and the overall SD classifier (right) are visualized as a boxplots in comparison with the true model mean $F_{0.5}$ score and a random guessing classifier. All tests did 1000 shuffles under 8-fold cross-validation, thus 8000 permutations each. Detailed result values presented in Table S8.

Table S8: Permutation test result values

classifier	permutations mean $F_{0.5}$ score	standard deviation	true model mean $F_{0.5}$ score	p-value
SD vs. C	0.42	0.076	0.86	< 0.001
SD vs. SR	0.48	0.047	0.85	< 0.001
SD vs. C & SR	0.07	0.055	0.88	< 0.001

Feature Inspection

Text S8. The shortlist of the most important (most discriminative) molecular features was generated using an importance scale of each feature's appearance in the last 10 (overall SD classification: last 12) features of each of the 136 independent recursive feature elimination processes. For example, if a molecular feature is mentioned 68 times in the last 10-feature list of the 136 models, its feature importance is calculated as 68 / 136 = 0.5. Based on this metric, the most important molecular features for each classification task were computed.

Where possible, we could provide identifications of molecular features that were confirmed by LC retention, MS1, and MS2 spectra using authentic reference standards. As with many other untargeted metabolomics studies, the identification of molecular features remains the most challenging part, attributed to the methodological drawback of limited fragment sensitivity, low-quality fragmentation spectra, and missing references in metabolome databases.[21] Nevertheless, for a future routine application of our metabolic fingerprint, these molecular features need to be isolated and unambiguously identified.

Table S9: Top-10 most important molecular features for SD vs. C classification according to the logistic regression model. The ones that are also indicative for SD vs. SR classification are highlighted in bold letters.

Rank	Molecular feature	Importance	Influence by sleep deprivation	Confirmed identity
1	8.33min_462.2195m/z_10431_HILIC+	0.81	increase	-
2	4.57min_448.2144m/z_13706_RP+	0.80	increase	-
3	**5.5min_966.4982m/z_19807_HILIC+**	0.74	decrease	-
4	4.47min_360.7081m/z_9174_RP+	0.65	increase	-
5	**7.63min_576.0182m/z_13505_HILIC+**	0.57	decrease	-
6	**5.63min_479.1852m/z_10894_HILIC+**	0.45	increase	-
7	10.47min_820.1332m/z_31345_RP+	0.43	increase	-
8	3.51min_218.1034m/z_1392_HILIC-	0.40	decrease	pantothenic acid
9	4.63min_259.1025m/z_3951_HILIC+	0.32	increase	AICA-riboside
10	8.14min_424.5774m/z_5114_HILIC-	0.31	decrease	-

Molecular feature name: retention time [underscore] mass-to-charge ratio [underscore] unique identifier [underscore] LC-MS condition
Monoisotopic masses uncertainty: +/- 20 ppm

Table S10: Top-10 most important molecular features for SD vs. SR classification according to the logistic regression model. The ones that are also indicative for SD vs. C classification are highlighted in bold letters.

Rank	Molecular feature	Importance	Influence by sleep deprivation	Confirmed identity
1	5.47min_304.2114m/z_5401_HILIC+	0.92	increase	3-hydroxyoctanoyl carnitine
2	1.79min_153.0678m/z_1354_HILIC+	0.88	decrease	nudifloramide
3	2.02min_116.0702m/z_639_HILIC+	0.68	increase	-
4	6.51min_619.7275m/z_23494_RP+	0.57	decrease	-
5	11.54min_673.0185m/z_26054_RP+	0.54	decrease	-
6	**5.5min_966.4982m/z_19807_HILIC+**	0.50	decrease	-
7	**5.63min_479.1852m/z_10894_HILIC+**	0.49	increase	-
8	11.55min_807.4209m/z_31032_RP+	0.46	decrease	-
9	5.63min_537.2374m/z_12500_HILIC+	0.40	increase	-
10	**7.63min_576.0182m/z_13505_HILIC+**	0.40	decrease	-

Molecular feature name: retention time [underscore] mass-to-charge ratio [underscore] unique identifier [underscore] LC-MS condition
Monoisotopic masses uncertainty: +/- 20 ppm

Table S11: Top-12 most important molecular features for SD vs. C and SR classification according to the logistic regression model. The ones that are also indicative for either SD vs. C or SD vs. SR classification are highlighted in bold letters.

Rank	Molecular feature	Importance	Influence by sleep deprivation	Confirmed identity
1	**5.47_304.2114m/z_5401_HILIC+**	0.86	increase	3-hydroxyoctanoyl carnitine
2	**1.79_153.0678m/z_1354_HILIC+**	0.82	decrease	nudifloramide
3	**5.63_479.1852m/z_10894_HILIC+**	0.74	increase	-
4+5	**2.02_116.0702m/z_639_HILIC+**	0.59	increase	-
	**11.54_673.0185m/z_26054_RP+**	0.59	decrease	-
6	6.07_378.1018m/z_4363_HILIC-	0.50	decrease	-
7	7.88_416.7819m/z_9080_HILIC+	0.47	increase	-
8	**5.63_537.2374m/z_12500_HILIC+**	0.45	increase	-
9	5.4_332.2202m/z_6388_HILIC+	0.37	decrease	-
10	**5.5_966.4982m/z_19807_HILIC+**	0.34	decrease	-
11+12	**7.63_576.0182m/z_13505_HILIC+**	0.33	decrease	-
	**4.63_259.1025m/z_3951_HILIC+**	0.33	increase	AICA-riboside

Molecular feature name: retention time [underscore] mass-to-charge ratio [underscore] unique identifier [underscore] LC-MS condition
Monoisotopic masses uncertainty: +/- 20 ppm

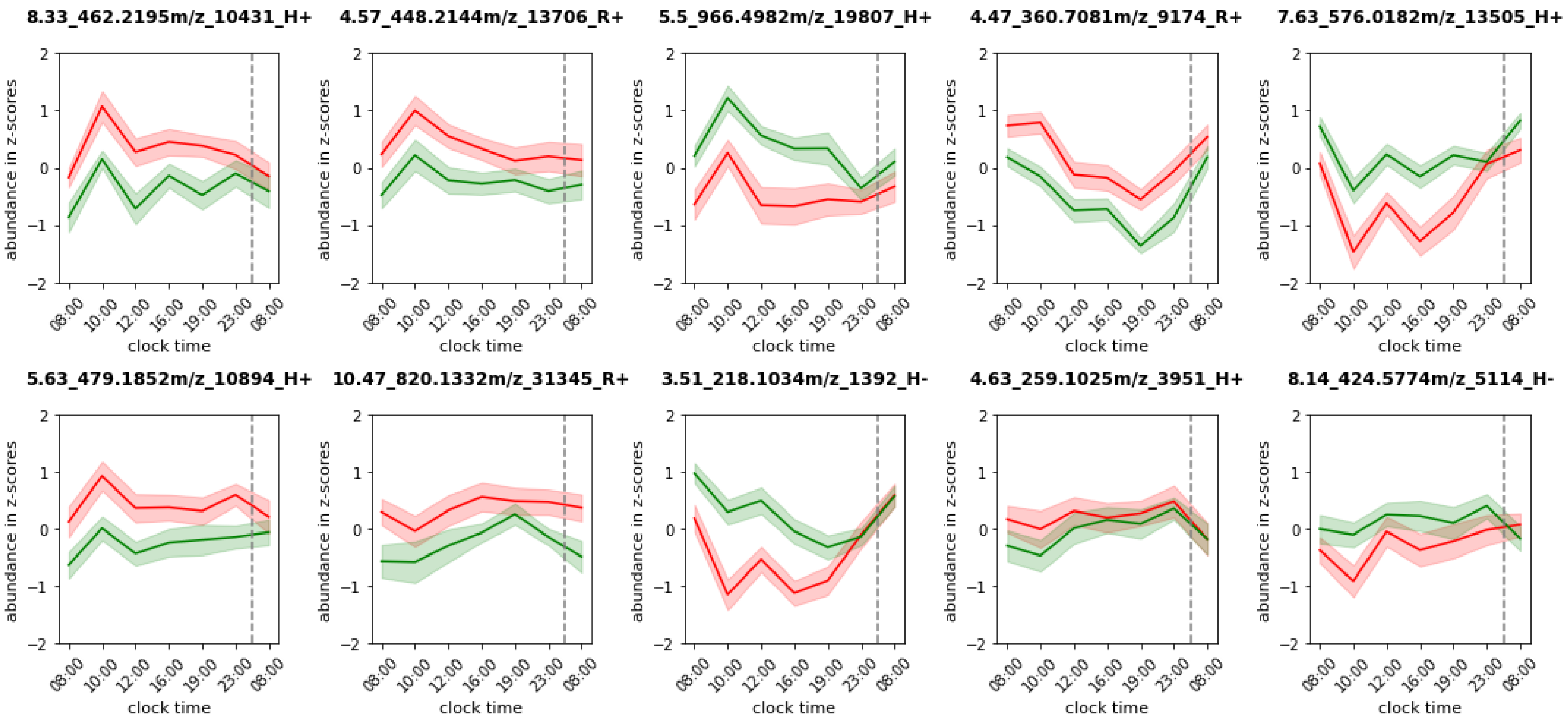

Figure S10: Top-10 most important molecular features of sleep deprivation (SD) vs. control (C) classification
Mean abundance in z-scores (y-axis) against clock time after intervention night (x-axis, not formatted to hour scale). Red lines represent SD intervention; green lines represent C intervention. Shades indicate the standard error of the respective mean of 20 participants. Dashed grey line between clock time 23:00 and 08:00 represents recovery night (end of intervention).

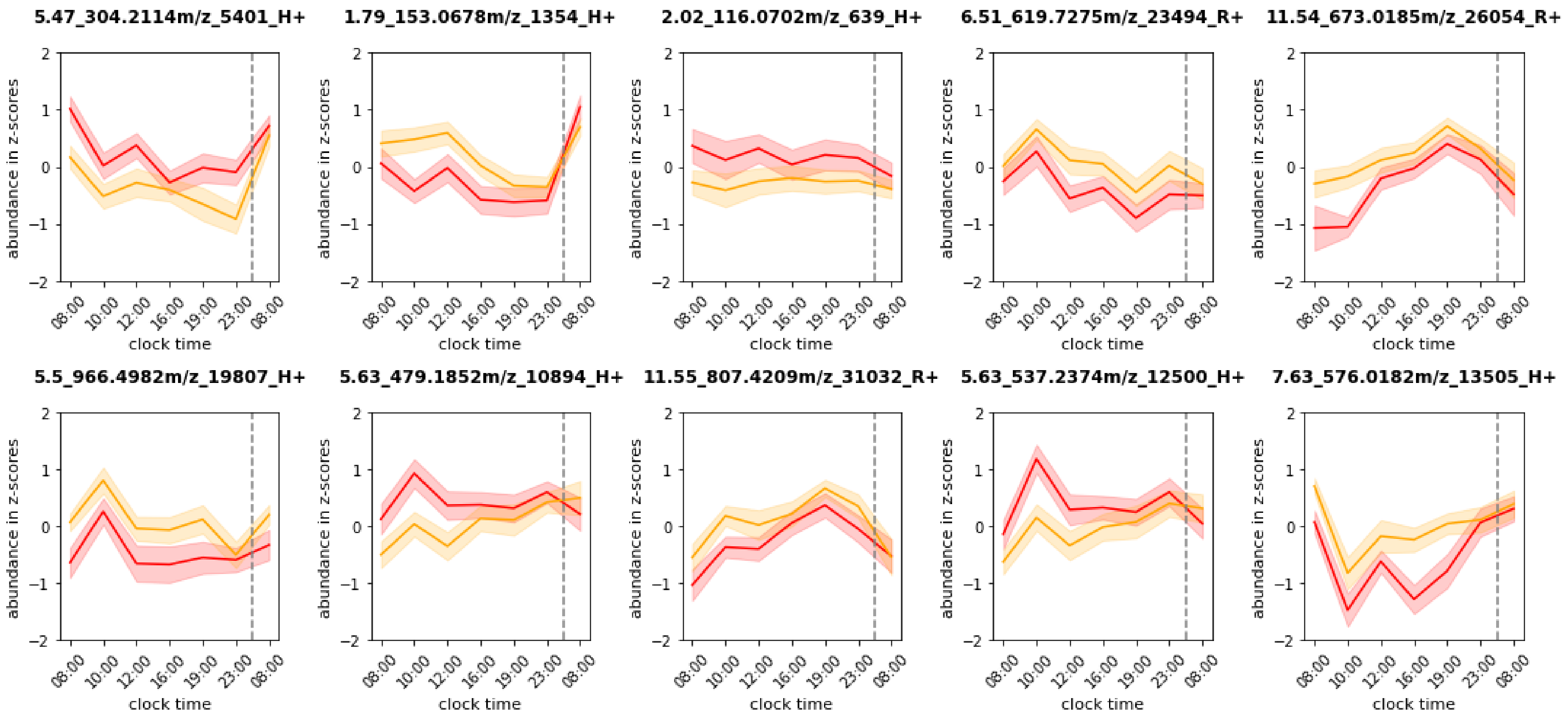

Figure S11: Top-10 most important molecular features of sleep deprivation (SD) vs. sleep restriction (SR) classification

Mean abundance in z-scores (y-axis) against clock time after intervention night (x-axis, not formatted to hour scale). Red lines represent SD intervention; orange lines represent SR intervention. Shades indicate the standard error of the respective mean of 20 participants. Dashed grey line between clock time 23:00 and 08:00 represents recovery night (end of intervention).

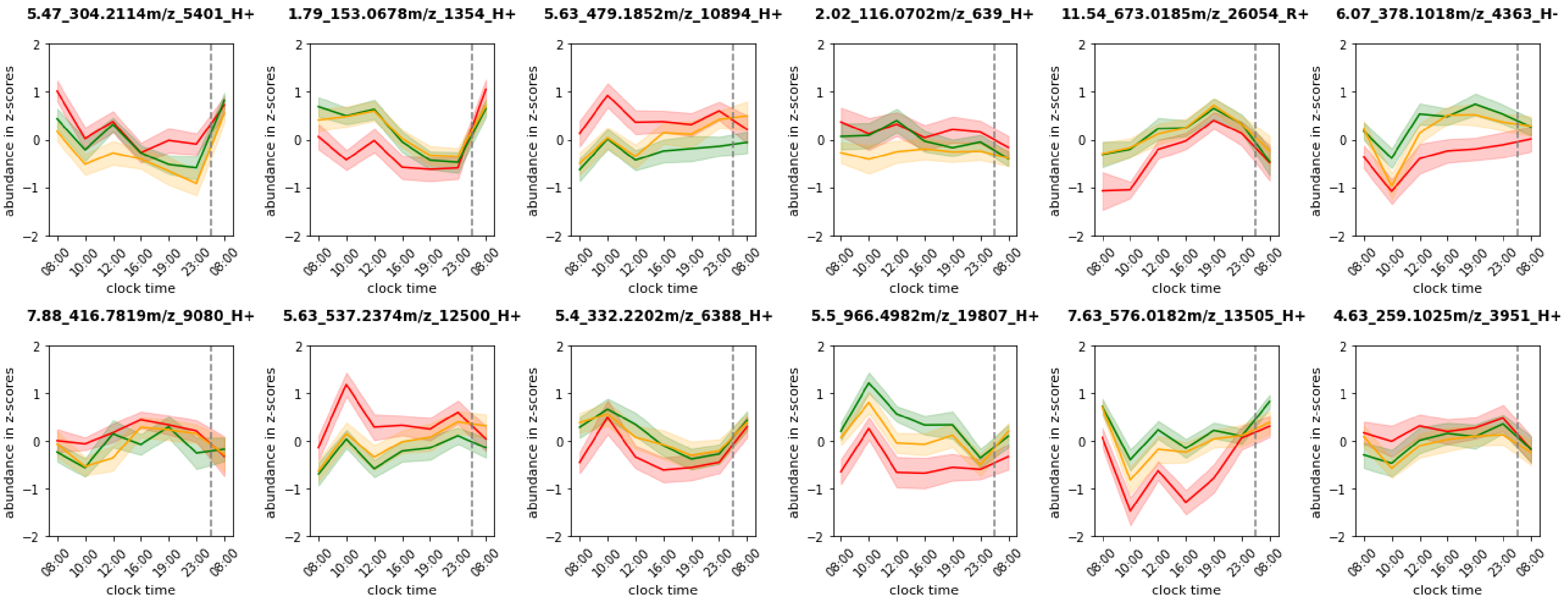

Figure S12: Top-12 most important molecular features of sleep deprivation (SD) vs. control (C) and sleep restriction (SR) classification

Mean abundance in z-scores (y-axis) against clock time after intervention night (x-axis, not formatted to hour scale). Red lines represent SD intervention; orange lines represent SR intervention; green lines represent C intervention. Shades indicate the standard error of the respective mean of 20 participants. Dashed grey line between clock time 23:00 and 08:00 represents recovery night (end of intervention).

Text S9. In the cosinor analysis, rhythmicity of the most important features was assessed by fitting a cosinor function to their DLMO-adjusted abundance measurements on both the individual and the population level, according to Cornelissen et al.[22] The goodness of fit to a 24-hour period model was evaluated by the significance of the fitting model (p value of F-test), the residual sum of squares (RSS) and its standard error (SE), the margin of error (ME), and the signal to noise (S/N) ratio, according to established works.[22,23] The significance level threshold was lowered to account for the number of measurements by the integreated calculate_significance_level function of CosinorPy.[24]
The results of the cosinor analysis are presented in Tables S12-S14 for the SD vs. C model features, the SD vs. SR model features, and the overall SD model features, respectively. Those features that most significantly follow a 24-hour period cosinor model from the overall SD classification model are visualized in Figure S13.

Table S12: Cosinor analysis results of top-10 most important molecular features for the SD vs. C classification model. Explanations and abbreviations see Text S9.

Rank	Molecular feature	Period [h]	p value	Significance level threshold	Model significance	RSS	SE	ME	S/N ratio
1	8.33_462.2195m/z_10431_H+	24	0.5627	0.0039	No	352.24	1.11	2.19	0.06
2	4.57_448.2144m/z_13706_R+	24	0.0423	0.0039	No	298.72	1.02	2.02	0.15
3	5.5_966.4982m/z_19807_H+	24	0.0206	0.0039	No	356.76	1.12	2.20	0.16
4	4.47_360.7081m/z_9174_R+	24	<0.0001	0.0039	Yes	303.55	1.03	2.03	0.27
5	7.63_576.0182m/z_13505_H+	24	<0.0001	0.0039	Yes	322.85	1.06	2.09	0.27
6	5.63_479.1852m/z_10894_H+	24	0.9553	0.0039	No	327.25	1.07	2.11	0.02
7	10.47_820.1332m/z_31345_R+	24	0.0177	0.0039	No	345.40	1.10	2.17	0.17
8	3.51_218.1034m/z_1392_H-	24	0.0011	0.0039	Yes	326.60	1.07	2.11	0.22
9	4.63_259.1025m/z_3951_H+	24	0.0615	0.0039	No	326.65	1.07	2.11	0.14
10	8.14_424.5774m/z_5114_H-	24	0.1890	0.0039	No	332.58	1.08	2.13	0.11

Table S13: Cosinor analysis results of top-10 most important molecular features for the SD vs. SR classification model. Explanations and abbreviations see Text S9.

Rank	Molecular feature	Period [h]	p value	Significance level threshold	Model significance	RSS	SE	ME	S/N ratio
1	5.47_304.2114m/z_5401_H+	24	0.0095	0.0040	No	318.07	1.07	2.11	0.18
2	1.79_153.0678m/z_1354_H+	24	<0.0001	0.0040	Yes	278.40	1.00	1.97	0.36
3	2.02_116.0702m/z_639_H+	24	0.8981	0.0040	No	311.32	1.06	2.09	0.03
4	6.51_619.7275m/z_23494_R+	24	0.3322	0.0040	No	319.82	1.07	2.12	0.09
5	11.54_673.0185m/z_26054_R+	24	<0.0001	0.0040	Yes	277.17	1.00	1.97	0.38
6	5.5_966.4982m/z_19807_H+	24	0.0602	0.0040	No	308.73	1.06	2.08	0.14
7	5.63_479.1852m/z_10894_H+	24	0.3806	0.0040	No	316.91	1.07	2.11	0.08
8	11.55_807.4209m/z_31032_R+	24	<0.0001	0.0040	Yes	251.27	0.95	1.87	0.39
9	5.63_537.2374m/z_12500_H+	24	0.6373	0.0040	No	328.37	1.09	2.14	0.06
10	7.63_576.0182m/z_13505_H+	24	<0.0001	0.0040	Yes	340.45	1.11	2.18	0.29

Table S14: Cosinor analysis results of top-12 most important molecular features for the SD vs. C and SR classification model. Explanations and abbreviations see Text S9.

Rank	Molecular feature	Period [h]	p value	Significance level threshold	Model significance	RSS	SE	ME	S/N ratio
1	5.47_304.2114m/z_5401_H+	24	0.0001	0.0025	Yes	456.43	1.03	2.03	0.21
2	1.79_153.0678m/z_1354_H+	24	<0.0001	0.0025	Yes	394.92	0.96	1.89	0.41
3	5.63_479.1852m/z_10894_H+	24	0.4050	0.0025	No	479.80	1.06	2.08	0.06
4	2.02_116.0702m/z_639_H+	24	0.5937	0.0025	No	449.41	1.02	2.01	0.05
5	11.54_673.0185m/z_26054_R+	24	<0.0001	0.0025	Yes	412.81	0.98	1.93	0.37
6	6.07_378.1018m/z_4363_H-	24	<0.0001	0.0025	Yes	404.33	0.97	1.91	0.23
7	7.88_416.7819m/z_9080_H+	24	0.0508	0.0025	No	441.52	1.01	1.99	0.12
8	5.63_537.2374m/z_12500_H+	24	0.2468	0.0025	No	483.47	1.06	2.09	0.08
9	5.4_332.2202m/z_6388_H+	24	<0.0001	0.0025	Yes	449.65	1.02	2.01	0.25
10	5.5_966.4982m/z_19807_H+	24	0.0008	0.0025	Yes	473.04	1.05	2.06	0.18
11	7.63_576.0182m/z_13505_H+	24	<0.0001	0.0025	Yes	462.69	1.04	2.04	0.26
12	4.63_259.1025m/z_3951_H+	24	0.0148	0.0025	No	462.49	1.04	2.04	0.14

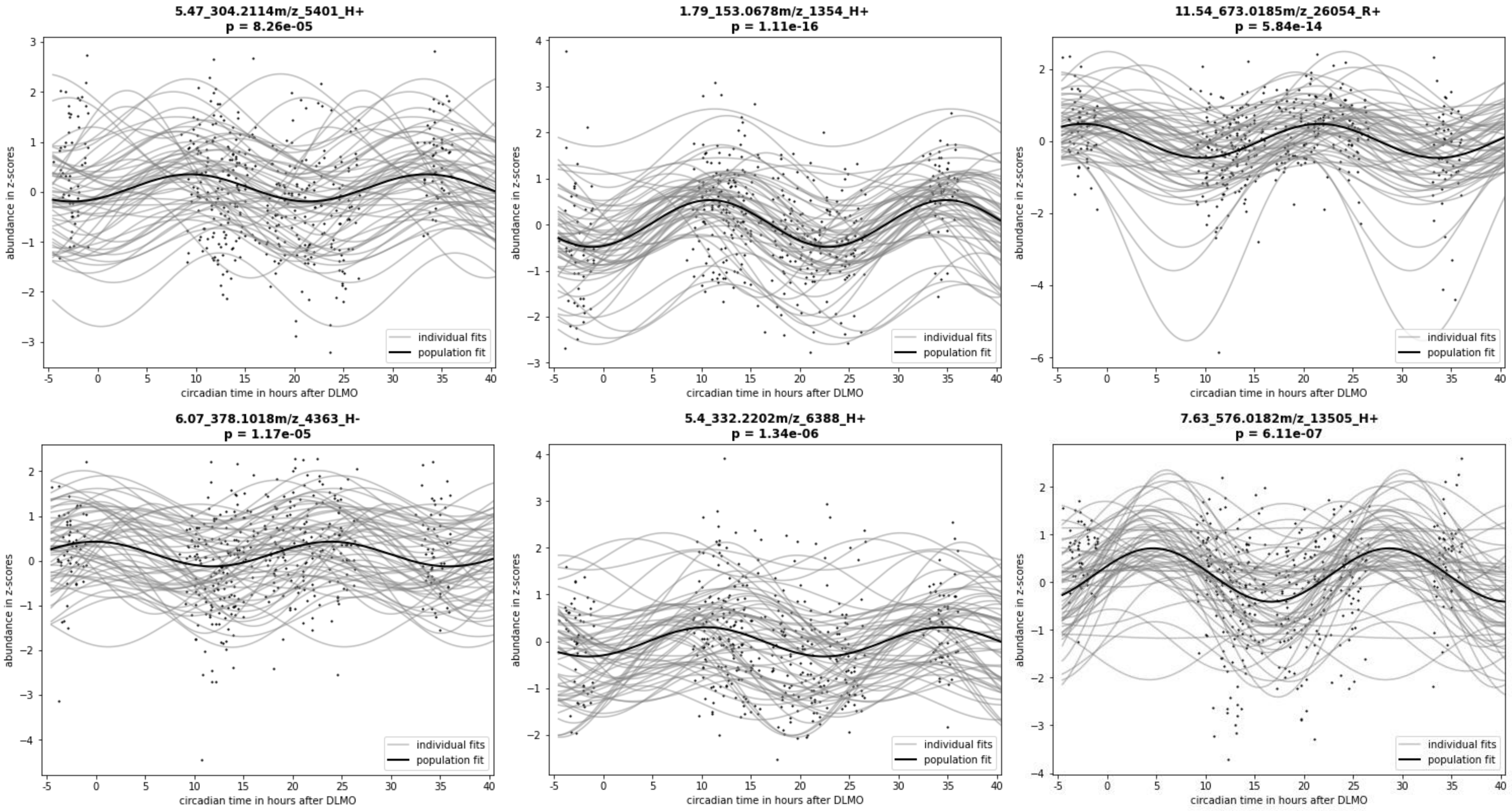

Figure S13: Cosinor model fittings of selected molecular features

Model significance for each feature quantified as p value of F-Test in titles. X-axis adjusted to individual dim-light melatonin onset (DLMO) time with DLMO time being 0 hours. Black scatters represent individual measurements, grey lines individual fits, and black lines population fit according to Cornelissen et al.

Additional Information

Chemicals, Reagents, and Materials

Text S10. Sarstedt AG (Sevelen, Switzerland) provided Salivette® sampling devices (with cotton swab No. 51.1534, with synthetic swab No. 51.1534.500). Methanol and acetonitrile (Optima® LC–MS grade), as well as ammonium acetate were acquired from Fisher Scientific (Basel, Switzerland), water of LC–MS grade from VWR (Dietikon, Switzerland). Formic acid (Ultra Liquid Chromatography-MS grade) was obtained from Biosolve (via Chemie Brunschwig AG, Basel, Switzerland). Dimethyl sulfoxide (DMSO) and acetone were delivered from Merck (Darmstadt, Germany). Compound reference standards of arginine, cortisol, cortisone, creatinine, glycocholic acid, hippuric acid, leucine, melatonin, raffinose, riboflavin, tryptophan, as well as ammonium formate, acetic acid, and ethyl acetate of LC-MS purity were acquired from Sigma-Aldrich (Buchs, Switzerland). Deuterated melatonin reference standard was purchased from Cayman Chemical (Ann Arbor, Michigan, USA). Isolute® SLE + columns were purchased from Biotage (Uppsala, Sweden). Eppendorf tubes were provided by Eppendorf SE (Hamburg, Germany), while conical HPLC vials were supplied from infochroma ag (Goldau, Switzerland).

Abbreviations

AUROC	Area under the ROC curve
C	Control [study intervention]
CV	Cross-validation
DDA	Data-dependent acquisition
DLMO	Dim-light melatonin onset
DMSO	Dimethyl sulfoxide
ESI	Electrospray ionization
h	Hour
HILIC	Hydrophilic interaction chromatography
LC	Liquid chromatography
LR	Logistic regression [classifier]
MCC	Matthews correlation coefficient
ME	Margin of error
MF	Molecular feature
ML	Machine learning
MS	Mass spectrometry
MS/MS	Tandem mass spectrum/spectrometry
QC	Quality control
QTOF	Quadrupole-time-of-flight
RFE	Recursive feature elimination
ROC	Receiver operator characteristic
RP	Reversed-phase
RSS	Residual sum of squares
S/N	Signal to noise [ratio]
SD	Sleep deprivation [study intervention]
SE	Standard error
SR	Sleep restriction [study intervention]
UHPLC	Ultra high performance liquid chromatography

References

(1) Scholz, M.; Steuer, A. E.; Dobay, A.; Landolt, H.-P.; Kraemer, T. Assessing the Influence of Sleep and Sampling Time on Metabolites in Oral Fluid: Implications for Metabolomics Studies. *Metabolomics* **2024**, *20* (5), 97. https://doi.org/10.1007/s11306-024-02158-3.

(2) Rosa, R. R.; Bonnet, M. H.; Warm, J. S. Recovery of Performance During Sleep Following Sleep Deprivation. *Psychophysiology* **1983**, *20* (2), 152–159. https://doi.org/10.1111/j.1469-8986.1983.tb03281.x.

(3) Peng, Z.; Hou, Y.; Xu, L.; Wang, H.; Wu, S.; Song, T.; Shao, Y.; Yang, Y. Recovery Sleep Attenuates Impairments in Working Memory Following Total Sleep Deprivation. *Front Neurosci* **2023**, *17*, 1056788. https://doi.org/10.3389/fnins.2023.1056788.

(4) Adams, K. J.; Pratt, B.; Bose, N.; Dubois, L. G.; St John-Williams, L.; Perrott, K. M.; Ky, K.; Kapahi, P.; Sharma, V.; MacCoss, M. J.; Moseley, M. A.; Colton, C. A.; MacLean, B. X.; Schilling, B.; Thompson, J. W.; Alzheimer's Disease Metabolomics Consortium. Skyline for Small Molecules: A Unifying Software Package for Quantitative Metabolomics. *J Proteome Res* **2020**, *19* (4), 1447–1458. https://doi.org/10.1021/acs.jproteome.9b00640.

(5) Boxler, M. I.; Schneider, T. D.; Kraemer, T.; Steuer, A. E. Analytical Considerations for (Un)-Targeted Metabolomic Studies with Special Focus on Forensic Applications. *Drug Test Anal* **2019**, *11* (5), 678–696. https://doi.org/10.1002/dta.2540.

(6) Steuer, A. E.; Arnold, K.; Schneider, T. D.; Poetzsch, M.; Kraemer, T. A New Metabolomics-Based Strategy for Identification of Endogenous Markers of Urine Adulteration Attempts Exemplified for Potassium Nitrite. *Anal Bioanal Chem* **2017**, *409* (26), 6235–6244. https://doi.org/10.1007/s00216-017-0567-4.

(7) Sands, C. J.; Wolfer, A. M.; Correia, G. D. S.; Sadawi, N.; Ahmed, A.; Jiménez, B.; Lewis, M. R.; Glen, R. C.; Nicholson, J. K.; Pearce, J. T. M. The nPYc-Toolbox, a Python Module for the Pre-Processing, Quality-Control and Analysis of Metabolic Profiling Datasets. *Bioinformatics* **2019**, *35* (24), 5359–5360. https://doi.org/10.1093/bioinformatics/btz566.

(8) Dunn, W. B.; Broadhurst, D.; Begley, P.; Zelena, E.; Francis-McIntyre, S.; Anderson, N.; Brown, M.; Knowles, J. D.; Halsall, A.; Haselden, J. N.; Nicholls, A. W.; Wilson, I. D.; Kell, D. B.; Goodacre, R. Procedures for Large-Scale Metabolic Profiling of Serum and Plasma Using Gas Chromatography and Liquid Chromatography Coupled to Mass Spectrometry. *Nat Protoc* **2011**, *6* (7), 1060–1083. https://doi.org/10.1038/nprot.2011.335.

(9) Broadhurst, D.; Goodacre, R.; Reinke, S. N.; Kuligowski, J.; Wilson, I. D.; Lewis, M. R.; Dunn, W. B. Guidelines and Considerations for the Use of System Suitability and Quality Control Samples in Mass Spectrometry Assays Applied in Untargeted Clinical Metabolomic Studies. *Metabolomics* **2018**, *14* (6). https://doi.org/10.1007/s11306-018-1367-3.

(10) Sangster, T.; Major, H.; Plumb, R.; Wilson, A. J.; Wilson, I. D. A Pragmatic and Readily Implemented Quality Control Strategy for HPLC-MS and GC-MS-Based Metabonomic Analysis. *Analyst* **2006**, *131* (10), 1075–1078. https://doi.org/10.1039/b604498k.

(11) Lewis, M. R.; Pearce, J. T. M.; Spagou, K.; Green, M.; Dona, A. C.; Yuen, A. H. Y.; David, M.; Berry, D. J.; Chappell, K.; Horneffer-van der Sluis, V.; Shaw, R.; Lovestone, S.; Elliott, P.; Shockcor, J.; Lindon, J. C.; Cloarec, O.; Takats, Z.; Holmes, E.; Nicholson, J. K. Development and Application of Ultra-Performance Liquid Chromatography-TOF MS for Precision Large Scale Urinary Metabolic Phenotyping. *Anal Chem* **2016**, *88* (18), 9004–9013. https://doi.org/10.1021/acs.analchem.6b01481.

(12) Ivanisevic, J.; Want, E. J. From Samples to Insights into Metabolism: Uncovering Biologically Relevant Information in LC-HRMS Metabolomics Data. *Metabolites* **2019**, *9* (12), E308. https://doi.org/10.3390/metabo9120308.

(13) Dieterle, F.; Ross, A.; Schlotterbeck, G.; Senn, H. Probabilistic Quotient Normalization as Robust Method to Account for Dilution of Complex Biological Mixtures. Application in 1H NMR Metabonomics. *Anal Chem* **2006**, *78* (13), 4281–4290. https://doi.org/10.1021/ac051632c.

(14) Gong, Y.; Ding, W.; Wang, P.; Wu, Q.; Yao, X.; Yang, Q. Evaluating Machine Learning Methods of Analyzing Multiclass Metabolomics. *J Chem Inf Model* **2023**, *63* (24), 7628–7641. https://doi.org/10.1021/acs.jcim.3c01525.

(15) Pedregosa, F.; Varoquaux, G.; Gramfort, A.; Michel, V.; Thirion, B.; Grisel, O.; Blondel, M.; Prettenhofer, P.; Weiss, R.; Dubourg, V.; Vanderplas, J.; Passos, A.; Cournapeau, D.; Brucher, M.; Perrot, M.; Duchesnay, É. Scikit-Learn: Machine Learning in Python. *JMLR* **2011**, *12* (85), 2825–2830.

(16) Hrydziuszko, O.; Viant, M. R. Missing Values in Mass Spectrometry Based Metabolomics: An Undervalued Step in the Data Processing Pipeline. *Metabolomics* **2012**, *8* (1), 161–174. https://doi.org/10.1007/s11306-011-0366-4.

(17) Troyanskaya, O.; Cantor, M.; Sherlock, G.; Brown, P.; Hastie, T.; Tibshirani, R.; Botstein, D.; Altman, R. B. Missing Value Estimation Methods for DNA Microarrays. *Bioinformatics* **2001**, *17* (6), 520–525. https://doi.org/10.1093/bioinformatics/17.6.520.

(18) Bergstra, J.; Bengio, Y. Random Search for Hyper-Parameter Optimization. *JMLR* **2012**, *13* (null), 281–305.

(19) Bellman, R. E. *Adaptive Control Processes: A Guided Tour*; Princeton University Press, 1961. https://doi.org/10.1515/9781400874668.
(20) *Permutation, Parametric, and Bootstrap Tests of Hypotheses*, 3rd ed.; Good, P., Ed.; Springer: New York, 2005. https://doi.org/10.1007/0-387-27158-9_7.
(21) da Silva, R. R.; Dorrestein, P. C.; Quinn, R. A. Illuminating the Dark Matter in Metabolomics. *PNAS* **2015**, *112* (41), 12549–12550. https://doi.org/10.1073/pnas.1516878112.
(22) Cornelissen, G. Cosinor-Based Rhythmometry. *Theor Biol Med Model* **2014**, *11*, 16. https://doi.org/10.1186/1742-4682-11-16.
(23) Bingham, C.; Arbogast, B.; Guillaume, G. C.; Lee, J. K.; Halberg, F. Inferential Statistical Methods for Estimating and Comparing Cosinor Parameters. *Chronobiologia* **1982**, *9* (4), 397–439.
(24) Moškon, M. CosinorPy: A Python Package for Cosinor-Based Rhythmometry. *BMC Bioinformatics* **2020**, *21* (1), 485. https://doi.org/10.1186/s12859-020-03830-w.
